# Targeted metabolomics of *Gammarus pulex* following controlled exposures to selected pharmaceuticals in water

**DOI:** 10.1016/j.scitotenv.2016.03.181

**Published:** 2016-08-15

**Authors:** Cristian Gómez-Canela, Thomas H. Miller, Nicolas R. Bury, Romà Tauler, Leon P. Barron

**Affiliations:** a Department of Environmental Chemistry, IDAEA-CSIC, Jordi Girona 18-26, 08034 Barcelona, Catalonia, Spain; b Analytical & Environmental Sciences Division, Faculty of Life Sciences and Medicine, King's College London, 150 Stamford Street, London SE1 9NH, UK; c Diabetes and Nutritional Sciences, Faculty of Life Sciences and Medicine, King's College London, 150 Stamford Street, London SE1 9NH, UK

**Keywords:** Pharmaceuticals, LC-HRMS, *Gammarus pulex*, Aquatic toxicology, Metabolomics

## Abstract

The effects of pharmaceuticals and personal care products (PPCPs) on aquatic organisms represent a significant current concern. Herein, a targeted metabolomics approach using liquid chromatography-high resolution mass spectrometry (LC-HRMS) is presented to characterise concentration changes in 29 selected metabolites following exposures of aquatic invertebrates, *Gammarus pulex*, to pharmaceuticals. Method performance revealed excellent linearity (*R*^2^ > 0.99), precision (0.1–19%) and lower instrumental limits of detection (0.002–0.20 ng) for all metabolites studied. Three pharmaceuticals were selected representing the low, middle and high range of measured acute measured toxicities (of a total of 26 compounds). Gammarids were exposed to both the no-observed-adverse-effect-level (NOAEL) and the lowest-observed-adverse-effect-level (LOAEL) of triclosan (0.1 and 0.3 mg L^− 1^), nimesulide (0.5 and 1.4 mg L^− 1^) and propranolol (100 and 153 mg L^− 1^) over 24 h. Quantitative metabolite profiling was then performed. Significant changes in metabolite concentrations relative to controls are presented and display distinct clustered trends for each pharmaceutical. Approximately 37% (triclosan), 33% (nimesulide) and 46% (propranolol) of metabolites showed statistically significant time-related effects. Observed changes are also discussed with respect to internal concentrations of the three pharmaceuticals measured using a method based on pulverised liquid extraction, solid phase extraction and LC-MS/MS. Potential metabolic pathways that may be affected by such exposures are also discussed. This represents the first study focussing on quantitative, targeted metabolomics of this lower trophic level benthic invertebrate that may elucidate biomarkers for future risk assessment.

## Highlights

• Metabolites of *Gammarus pulex* were determined following liquid chromatography coupled high resolution mass spectrometry.
• The toxicity study of 26 pharmaceuticals in *Gammarus pulex* show values between 0.57 mg L^− 1^ to > 250 mg L^− 1^.
• Variations in the metabolite concentrations were detected in the pharmaceuticals exposed samples respect to control samples.
• Pathway alterations related to protein synthesis, oxidative stress and signaling cascades were observed in exposed samples.

## Graphical abstract

**Figure:**
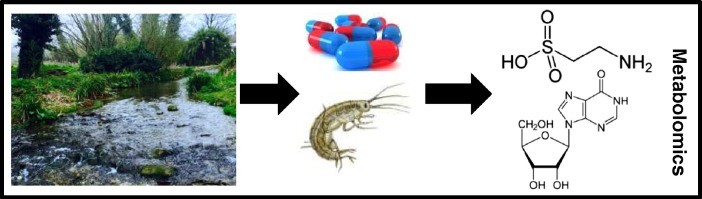

## 1 Introduction

In the last decade, pharmaceuticals have been recognised as an emerging class of environmental contaminants and their fate, occurrence and physicochemical behaviour in the aquatic environment have been extensively studied and reviewed (Daughton and Ternes, 1999, Evgenidou et al., 2015, Monteiro and Boxall, 2010). The ecotoxicological consequences of incomplete removal of pharmaceuticals or their metabolites in wastewater or drinking water treatment plants (WWTP/DWTP) are a matter of current environmental concern which still requires further research (Gómez-Canela et al., 2013). Furthermore, pharmaceuticals designed for hospital use are suspected to have more adverse effects than other pharmaceuticals regarding their effect on the aquatic environment (Gómez-Canela et al., 2014, Franquet-Griell et al., 2015). As such, in the last decade, much research has focused on understanding the occurrence and effects (Heberer, 2002) of these contaminants in exposed organisms. Recently, we developed a multi-residue analytical method and determined occurrence of pharmaceuticals in tributaries of the River Thames and in *Gammarus pulex* (*G. pulex*) at ng L^− 1^ and ng g^− 1^ concentrations respectively across eight sites (Miller et al., 2015). *G. pulex* is a small, low trophic level species of amphipod crustacean found in freshwaters across Europe and is very common throughout the United Kingdom (UK). *G. pulex* has many attributes for use in biomonitoring studies including its important role in freshwater food chains where they serve as a food source for other invertebrates, fish and birds (Maltby et al., 2002). This organism has also been extensively used in contaminant monitoring including toxicity assays for various pollutants such as metals, pharmaceuticals, PAHs/PCBs and natural stressors which has shown the importance of this species in environmental risk assessment (Bourgeault et al., 2013, Coulaud et al., 2011, De Lange et al., 2006, De Lange et al., 2009, Lebrun et al., 2012, Maltby et al., 2002, Pellet et al., 2014, Schaller et al., 2011, Vellinger et al., 2012).

Studies of the effects of pharmaceuticals on low trophic level invertebrate organisms such as *G. pulex* using high resolution confirmatory chemical analysis techniques are lacking. In particular, metabolomics may reduce this knowledge gap by directing effect-based studies that reveal alternative markers or end-points to assess potential toxicity of contaminants. Advances in mass spectrometry (MS) over the last decade have enabled better characterization of the links between the metabolome and phenotype (Dettmer et al., 2007, Villas-Bôas et al., 2005). Targeted liquid chromatography coupled to mass spectrometry (LC-MS) is the technique of choice for the reliable quantitation of known, pre-selected metabolites. It is an approach that will increasingly be used to apply knowledge discovered by non-targeted metabolomics; i.e. the eventual targeted measurement of a metabolic biomarker signature that can predict exposure to a specific environmental stress (Viant, 2008, Viant and Sommer, 2013). Metabolomics studies have made use of high resolution, confirmatory analytical techniques such as nuclear magnetic resonance (NMR) or hyphenated MS technologies to characterise large numbers of compounds for metabolic profiling (targeted) (Zhang et al., 2012). With the development of high resolution mass spectrometry (HRMS), non-targeted screening of several thousand compounds has become possible for studying larger portions of the metabolome in contrast to NMR, which is often limited by low sensitivity (Dunn et al., 2005, Zhang et al., 2012). Early investigations focused on human, plant and microbial metabolomes (Frisvad and Filtenborg, 1983, Horning and Horning, 1971, Taylor et al., 2002, Tweeddale et al., 1998). Other studies have identified changes in metabolomic profiles in mussels resulting from hypoxic conditions; biomarkers associated with withering syndrome in abalone sea snails; and responses to ethinylestradiol (EE2) by rainbow trout (Hines et al., 2007, Samuelsson et al., 2006, Viant et al., 2003). Approaches to characterise the metabolome can involve non-targeted or targeted strategies where for quantitative purposes, targeted analysis offers greater accuracy and precision (Griffiths et al., 2010, Lei et al., 2011).

The aim of the present study was to develop a targeted multi-residue method for the determination of 29 metabolites pertaining to different biochemical classes (amino acids, organic acids, nucleosides, nucleotides, and sugars) using LC-HRMS. As a full scan method also suitable for non-target analysis, the analytical performance of the method was evaluated quantitatively in terms of comprehensive mass spectral characterization, selectivity, sensitivity, intra- and inter-day precision, range and linearity. Acute toxicity of 26 pharmaceuticals in *G. pulex* was also assessed as an initial screen for compound selection for metabolomics. Three pharmaceuticals showing low, median and high measured LC_50_ values were then selected for exposures at no-observed-adverse-effect-level (NOAEL) and lowest-observed-adverse-effect-level (LOAEL) concentrations to evaluate alterations in the metabolite profile at 2, 6 and 24 h. This represents the first environmental metabolomics-based investigation of *G. pulex* by determining changes in endogenous metabolites in response to pharmaceutical residue exposure.

## 2 Materials and methods

### 2.1 Reagents, chemicals and consumables

All pharmaceuticals were purchased from Sigma-Aldrich (Steinheim, Germany) and Fluka (Buchs, Switzerland) with a purity of ≥ 97%. HPLC grade acetone, dimethylsulfoxide (DMSO), ethanol (EtOH) and methanol (MeOH) were purchased from Fischer Scientific (Loughborough, UK). Ultra-pure water was sourced from a Millipore Milli-Q water purification system with a specific resistance of 18.2 MΩ cm or greater (Millipore, Bedford, MA, USA). Stock solutions (40 mg mL^− 1^) were prepared in ultrapure water, acetone, MeOH, EtOH or DMSO, respectively. All stock solutions were stored in silanised amber vials (40 mL) and at − 20 °C in the dark for optimum stability. Organic solvent concentration (acetone, MeOH, EtOH and DMSO) in aqueous solutions used for toxicity testing was negligible. Metabolite standards (organic acids, nucleosides, nucleotides, sugars and amino acids) were supplied by Sigma Aldrich (Steinheim, Germany) and Fluka (Buchs, Switzerland). In addition, the isotopically-labelled algal amino acid mixture (98 atom% as ^13^C, 98 atom% as ^15^N) was provided from Sigma Aldrich. The targeted metabolome studied was comprised of 15 amino acids (AAs), 4 nucleosides, 2 nucleotides, 1 sugar, 3 organic acids and 4 compounds related to other families. Full details of the target metabolites and the labelled compounds are shown in Table 1. Finally, the 26 pharmaceutical compounds belonging to 12 different therapeutic classes are listed in Table S1.

### 2.2 Sample collection and preparation

Adult *G. pulex* were collected several times between September 2014 and April 2015 from the River Cray, UK, a tributary of the River Darent that feeds into the River Thames (51°23′10.5″N 0°06′34.8″E). Adult specimens were collected via the kick sampling netting method. Samples were transported back to the laboratory in Nalgene™ flasks containing a 500 mL grab sample of freshwater obtained from the River Cray. This site has previously been demonstrated to have low pharmaceutical contamination (< LOQ) (Miller et al., 2015). After collection, *G. pulex* were stored in different artificial freshwater tanks and acclimatized for a minimum of 2–3 days at 15 ± 2 °C under a 12 h:12 h light:dark cycle to allow depuration of any residual contamination. The freshwater crustaceans were fed ad libitum with a minimum of three horse-chestnut leaf discs (Ashauer et al., 2011). Artificial freshwater (AFW) was prepared following United States Environmental Protection Agency (USEPA) regulation (USEPA, 2002). Briefly, 1.20 g MgSO_4_, 1.92 g NaHCO_3_ and 0.080 g KCl were added to 19 L of ultra-pure water that was aerated overnight. In parallel, 1.20 g of CaSO_4_·2H_2_O was added to 1 L of Milli-Q water and mixed with the previous salt solution to make a total 20 L of AFW (USEPA, United States Environmental Protection Agency USEPA, 2002).

### 2.3 Pharmaceutical exposures

To select specific pharmaceuticals, the concentrations for exposures and subsequent metabolite profiling, a series of acute toxicity tests to 26 pharmaceuticals were initially performed following the Organization for Economic Co-operation and Development (OECD) 1488/94 guideline (European Communities Commission, 1996). Lethal median concentration effects and its 95% confidence interval (CI), were estimated by fitting immobility concentration responses to the Hill regression model (Eq. 1).

(1) $$I\left(C_{i}\right)=\frac{1}{1+{\left(\frac{C_{i}}{LC_{50}}\right)}^{-\mathrm{Hill}}}$$

Where, *I*(*C*_i_) is the proportion of immobile animals at concentration *C*_i_; *C*_i_ is the concentration of the respective compound (i); LC_50_ is the median lethal concentration to the 50% of population and *Hill* is the shape constant, which depends on the parameters adjusted in the regression model. From these toxicity profiles the NOAEL and the LOAEL were determined. Exposure experiments were performed in Pyrex® beakers, each containing 200 mL of AFW at 15 °C and ten adult animals (> 5 mg wet weight, ww). Live/dead animals were counted after 24 h by gently prodding and observing movement of appendages. The pharmaceutical concentrations tested were in the range of 0.01 to 250 mg L^− 1^. Control (AFW only) and solvent controls showed no measurable toxicity. Concentrations where 100, 50 and 0% of the animals died were repeated in duplicate or triplicate.

From the initial toxicity tests on 26 pharmaceuticals, 3 were chosen for metabolomics studies representing the high (triclosan), medium (nimesulide) and low (propranolol) range of measured acute toxicity. Each exposure consisted of *G. pulex* exposed at the NOAEL (*C*_1_) and a second higher concentration at the LOAEL (*C*_2_) as follows: triclosan, *C*_1_ = 0.1 mg L^− 1^/*C*_2_ = 0.3 mg L^− 1^; nimesulide, *C*_1_ = 0.5 mg L^− 1^/C_*2*_ = 1.4 mg L^− 1^; and propranolol, *C*_1_ = 100 mg L^− 1^/C_2_ = 153 mg L^− 1^. Approximately 100 specimens were introduced in a tank containing 1 L of AFW (control), 1 L of AFW spiked with *C*_1_ and 1 L of AFW with C_2_. At three different times (2, 6 and 24 h), 4 replicates of live *G. pulex* (a pool of 6–7 animals for each replicate) were collected, frozen on dry ice and then stored at − 80 °C before metabolite profiling was performed. A preliminary extraction protocol with 1, 3, 6 and 10 animals showed that a pool of 6–7 *G. pulex* provided the best method recoveries.

Quantification of pharmaceutical concentrations in *G. pulex* at the 24 h exposure interval was performed. Separate exposures were set up in triplicate in beakers containing 200 mL of exposure solution (AFW spiked with the respective *C*_1_ pharmaceutical dose) and 20 organisms. At 24 h, animals were immediately rinsed with ultra-pure water and then frozen at − 20 °C for 24 h. Full analytical method details are described in Miller et al., 2015. Prior to extraction, frozen *G. pulex* samples were lyophilised at − 50 °C under vacuum for 48 h and milled in an agate mortar to a fine powder. For each analysis, 20 mg of a lyophilised composite sample were transferred to 2 mL polypropylene tubes (Eppendorf®, Hamburg, Germany), for solid-liquid extraction (SLE). After the addition of 80 μL of stable isotope-labelled internal standards at concentrations between 3.29 and 43.11 ng μL^− 1^ (final concentration dependent on the AA concentration in the stock reference material mixture), 800 μL of MeOH:HPLC water (90:10) mixture was added and the samples were thoroughly mixed using a Vortex mixer. Then, samples were shaken for 25 min on a vibration plate (IKA® KS 260 basic) and centrifuged at 14,000 rpm for 25 min at 0 °C. Finally, the supernatant was transferred to a chromatographic vial by using a 0.20 μM syringe filter (Whatman, GE Healthcare Life Sciences, Buckinghamshire, UK). All samples were stored at − 80 °C until LC-HRMS analysis.

### 2.4 Internal pharmaceutical residue determination and metabolite profiling

Internal concentrations of pharmaceuticals in *G. pulex* were determined using a previously described method (Miller et al., 2015). Briefly, 50 mg of lyophilised *G. pulex* were extracted in 5 mL of acetonitrile and pre-concentrated on Oasis HLB SPE cartridges (6 mL, 200 mg sorbent). The extract was eluted, dried-down and reconstituted in starting LC mobile phase. The chromatographic separation followed a 75 min gradient (including 12.5 min re-equilibration) using water and acetonitrile with 10 mM ammonium acetate salt. Separation was achieved using a Waters Sunfire C_18_ reversed-phase column (2.1 × 150 mm, 2.5 μM particle size) and detection was performed by a Waters Quattro triple quadrupole mass analyser using positive and negative electrospray ionization polarity switching (Waters Corporation, Milford, MA, USA). Organic acids, nucleosides, nucleotides, sugars and AAs were measured using LC–HRMS. An Exactive™ mass spectrometer equipped with heated electrospray ionization (H-ESI) source was used (Thermo Fisher Scientific, Bremen, Germany). The system was equipped with a HTC PAL autosampler and a Surveyor MS Plus pump. A TSK Gel-Amide 80 column (2 × 250 mm, 5 μm) for analyte separation was purchased from Sigma Aldrich. Mobile phases were binary mixtures of acetonitrile (A) and 5 mM ammonium acetate (pH 5) in HPLC-grade water (B). Gradient elution started at 75% A and 25% B, which was increased linearly to 30% B in 8 min, increased linearly to 60% B to 12 min and then held for a further 5 min. Initial conditions were returned in 3 min and the system was stabilized after a total equilibration time of 10 min (total run time = 30 min). The flow rate was set at 150 μL min^− 1^ and the injection volume was 5 μL. Metabolites were analysed under positive/negative ESI mode, but better resolution was obtained in negative ionization mode and so this was used for all experiments. Full scan acquisition over a mass range of 80–800 Da was performed at 70,000 full width at half maximum (FWHM) and spray voltage at 3.00 kV, capillary voltage at 30 V, skimmer voltage at 28 V and tube lens voltage at 130 V were used. A sheath gas flow rate of 45 arbitrary units (au), an auxiliary gas flow rate of 10 au and a capillary temperature at 300 °C were selected.

### 2.5 Method performance and quantification

Method performance for internal pharmaceutical residue concentrations in *G. pulex* are described elsewhere (Miller et al., 2015). Here, pharmaceutical residue quantification in exposed *G. pulex* was performed by a 3-point matrix-matched calibration curve. For nimesulide, spiking concentrations were 2.5, 5 and 10 μg g^− 1^; for triclosan, these were 1, 2.5 and 5 μg g^− 1^; and for propranolol these were 1, 2.5 and 5 mg g^− 1^. The calibration range for endogenous metabolites was from 0.01 to 15 ng μL^− 1^, using 9 calibration points. The algal amino acid mixture-^13^C,^15^N (see Table 1) was used as internal standard (IS) for extraction and analytical control. l-ornithine hydrochloride, inosine, thymidine, taurine, 1.7-dimethylxanthine and (−)-riboflavin were quantified using either ^13^C,^15^N-proline or ^13^C,^15^N-tyrosine as the internal standard. The remaining 15 compounds were quantified using external calibration and the target compound itself was used as external standard. The instrumental detection limit (IDL) was initially calculated as that concentration giving a signal intensity of 1 × 10^3^, and afterwards measured experimentally by injecting a standard concentration that gave this signal intensity. The method detection limit (MDL) was calculated following the same procedure, using spiked lyophilised *G. pulex* samples at a concentration of 1 μg g^− 1^. Intra-assay variation was assessed using five consecutive injections of 5 ng μL^− 1^ standard solution, and inter-assay variation was determined by measuring the same standard solution on four different days. Solvent blanks did not contain any of the investigated analytes, indicating no carry-over between LC-HRMS runs. Recovery studies were performed in triplicate, using lyophilised *G. pulex* samples spiked at 1 μg g^− 1^ with the metabolites mixture and the algal amino acid mixture-^13^C, ^15^N. Five replicates of a pool with 6–7 *G. pulex* were analysed first and the traces of target compounds were subtracted.

### 2.6 Statistical analysis

Two-way ANOVA considering significant *p* values ≤ 0.05 was used as a first step to select metabolites with significant changes and to further explore their concentration and time trends. Thus, *p* values were derived and examined to determine any differences between exposed and control specimens, and to evaluate the effect of the exposure time. All data satisfied the assumptions of normality and homoscedasticity. In addition, all calculations were performed in MATLAB software version R2013b.

## 3 Results and discussion

### 3.1 Analytical method performance

**Fig. 1:**
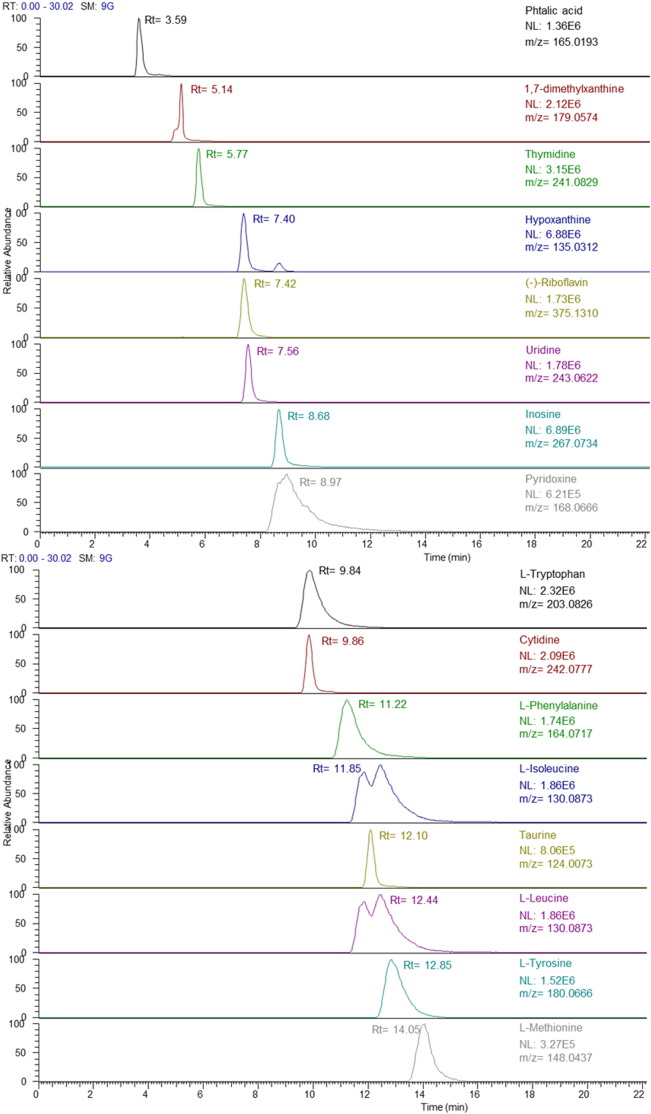

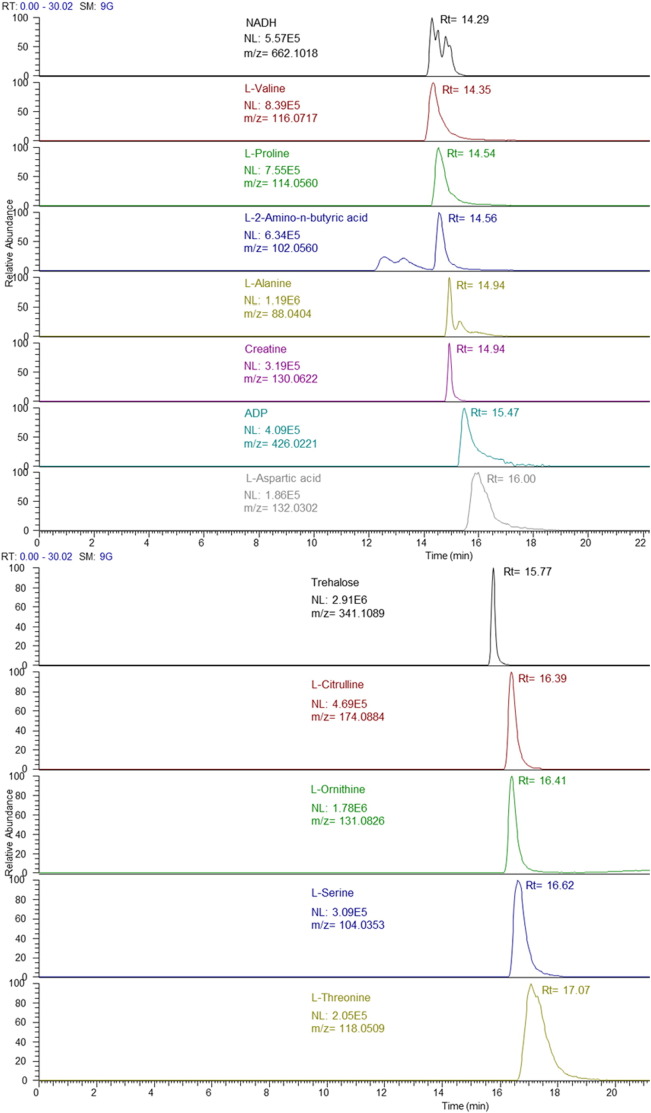
LC–HRMS extracted ion chromatogram of 29 target compounds from a full scan LC-Orbitrap-MS spectrum using a 5 ppm extraction window.

Good correlation coefficients (R^2^ ≥ 0.99) were obtained for 29 metabolites (Table 1). Responses for fifteen metabolites were linear from 0.05 to 15 ng μL^− 1^; and for 11 other metabolites, linearity ranged from 0.1 to 15 ng μL^− 1^. Signals for inosine and hypoxanthine were linear over the range of 0.01 to 15 ng μL^− 1^; and ADP in the range 3 to 15 ng μL^− 1^. Therefore, given that this represents a lower concentration range of 10 to 1500 ng g^− 1^, these were considered fit for purpose for this study. IDL ranged from 0.002 to 0.20 ng, and intra and inter-day precision ranges were from 1 to 14% and from 0.1 to 19%, respectively at the 5 ng μL^− 1^ concentration level. Twenty seven compounds showed recoveries within the range of 42 ± 8% to 138 ± 2% (Table 1). Overall, recoveries were acceptable and showed excellent precision. However, the two nucleotides ADP and NADH displayed poorer recoveries of 25 ± 11% and 162 ± 10%, respectively. Finally, the MDL ranged from 0.04 (inosine) to 37.3 ng g^− 1^ (creatine), with the exception of ADP, for which sensitivity was low as would be expected due to the lower recovery observed (MDL: 162 ng g^− 1^). The extracted ion chromatograms of a target metabolite mixed solution at 5 ng μL^− 1^ using the TSK Gel-Amide 80 HILIC column is shown in Fig. 1. In summary, the method performance of this analytical method indicated that reliable quantitative measurements could be made for most metabolites and with minimal variance contribution from the matrix. Moreover, as this method incorporated HRMS in full-scan mode, *post hoc* untargeted data analysis of the metabolome remains possible if required.

### 3.2 Acute toxicity, 24-h pharmaceutical exposures and observed changes in target metabolite concentrations

Acute toxicity tests revealed LC_50_ at the mg L^− 1^ level for most of the 26 tested pharmaceuticals. Similar values have been obtained in other freshwater crustaceans (see Table S3). Kim et al. (2009) studied the acute toxicity of pharmaceuticals and personal care products in the freshwater crustacean, *Thamnocephalus platyurus (T. platyurus)*. They reported a similar LC_50_ value to the present study for triclosan (0.57 mg L^− 1^), but not for propranolol which lay at 10.31 mg L^− 1^ (Kim et al., 2009). These values provide a means of ranking the toxic risk, but such values are not environmentally relevant as pharmaceuticals are generally found at concentrations approximately one to two orders of magnitude less (Miller et al., 2015). Therefore, it is unlikely that the majority of these compounds will display any significant acute toxicity. Nonetheless, as a starting point and based on these data, exposures were performed at NOAEL and LOAEL concentrations for triclosan, nimesulide and propranolol to represent compounds at the high, median and low range of measured LC_50_ (Figs. S1–S3).

#### 3.2.1 Triclosan

**Fig. 2:**
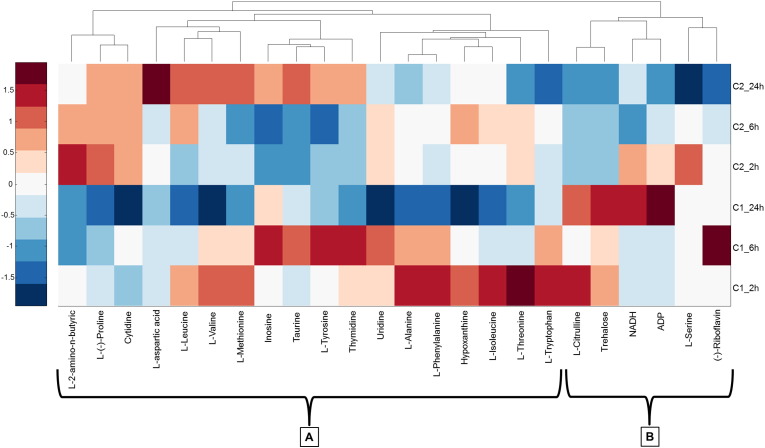
Heat map of triclosan exposure representing the fold change of targeted metabolites in each exposure subgroup (*C*_1_, *C*_2_) relative to controls. All data have been auto scaled by column and dendrograms represent hierarchical analysis for clustering (A & B) of metabolite responses.

Triclosan, the most toxic of the selected pharmaceuticals (LC_50_ 0.57 mg L^− 1^) measured here, caused changes in the metabolite concentrations in *G. pulex* at 0.1 mg L^− 1^ (*C*_1_) and 0.3 mg L^− 1^ (*C*_2_), and was dose responsive. Exposure to triclosan produced significant changes (Two-way ANOVA, *p* < 0.05) in 19 metabolites: 13 amino acids (l-alanine, cytidine, l-citrulline, l-isoleucine, l-leucine, l-methionine, l-phenylalanine, l-proline, taurine, l-threonine, l-tryptophan, l-tyrosine and l-valine), inosine and uridine (nucleosides) and others like trehalose, hypoxanthine, riboflavin and thymidine (Table 2 and Figure S4). However, only the concentration of 37% of metabolites changed significantly (up or down) across the exposure time. l-isoleucine, l-phenylalanine, l-(−)-proline, taurine, l-threonine, l-tyrosine, l-valine, inosine and thymidine concentrations varied significantly (*p* < 0.05) with exposure duration (Table 2). A 2-way ANOVA was used to evaluate the interaction between dose and time factors. Cytidine, l-methionine, l-phenylalanine and l-(−)-proline were the only metabolites that had significant interactions (Table 2). As shown in Figure S4, all metabolites except l-citrulline changed in their mean concentrations at *C*_1_ relative to the controls. Some metabolites increased more than two to three-fold in comparison to controls such as l-tryptophan, hypoxanthine, l-alanine, l-isoleucine, l-threonine, l-valine and l-thymidine. In other cases, measured concentrations between the exposed and control samples decreased over time (ADP and l-proline). Similar trends occurred at *C*_2_ where the 75% of the metabolites increased in their concentration, with the remaining metabolites showing no significant changes (also see Table S1). Table S2 shows the metabolite concentrations determined from all conditions studied. To more conveniently highlight trends, Fig. 2 represents the fold change values at each exposure concentration with respect to controls and plotted in a heat map and hierarchical analysis revealed two distinct clusters (A & B). In general, and for each metabolite in Cluster A of Fig. 2, the fold changes in gammarid metabolites exposed at *C*_1_ decreased across the 24-h period. On the contrary, Cluster B generally showed the opposite trend where concentrations of metabolites mostly increased. In the case of samples exposed at *C*_2_, eight metabolites of Cluster A (l-aspartic acid, l-leucine, l-valine, l-methionine, inosine, taurine, l-tyrosine and thymidine) had a fold increase and the remainder showed a slight decrease in concentration. The metabolites of Cluster B also showed fold decreases along the 24-h period and the opposite effect to those exposed at *C*_1_.

#### 3.2.2 Nimesulide

**Fig. 3:**
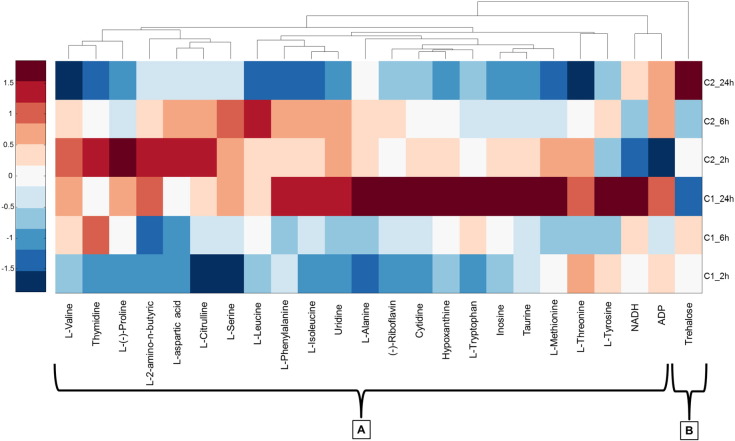
Heat map of nimesulide exposure representing the fold change of targeted metabolites in each exposure subgroup (*C*_1_, *C*_2_) relative to controls. All data have been auto scaled by column and dendrograms represent hierarchical analysis for clustering (A & B) of metabolite responses.

Exposure to nimesulide produced significant changes (Two-way ANOVA, *P* < 0.05) for 83% of the metabolites analysed, including most amino acids (except l-alanine), such as inosine, uridine and other metabolites including hypoxanthine, riboflavin and thymidine (Table 2 and Fig. S5). l-2-amino-*n*-butyric (AABA), l-alanine, l-aspartic acid, cytidine, l-citrulline, l-leucine, l-(−)-proline, l-serine, trehalose and thymidine showed a time-related effect (Table 2), affecting 42% of the metabolites studied. On the other hand, considering the interaction between time and dose factor, significant differences (*p* < 0.05) in all metabolites except for l-tyrosine and NADH were detected (Table 2). Nimesulide exposures induced changes in the metabolome of *G. pulex* in comparison to controls (Fig. S5). A heat map of nimesulide was prepared as before and hierarchical analysis again revealed two clusters (Fig. 3). In this case, at *C*_1_, all the metabolites except trehalose had fold increases along the 24-h period (Cluster A, Fig. 3). Specifically, l-alanine, (−)-riboflavin, cytidine, hypoxanthine, l-tryptophan, inosine, taurine, l-tyrosine and NADH suffered the more important changes (garnet colour). On the other hand, trehalose (Cluster B, Fig. 3) showed the opposite trend and, at 24-h exposure time, the fold change had decreased (blue). In the samples exposed at *C*_2_, the metabolites in Cluster A began with positive fold changes (red/orange) at 2-h exposure time and decreased (blue) at 24-h exposure time, with the exception of NADH, ADP and trehalose (Cluster B, Fig. 3).

#### 3.2.3 Propranolol

**Fig. 4:**
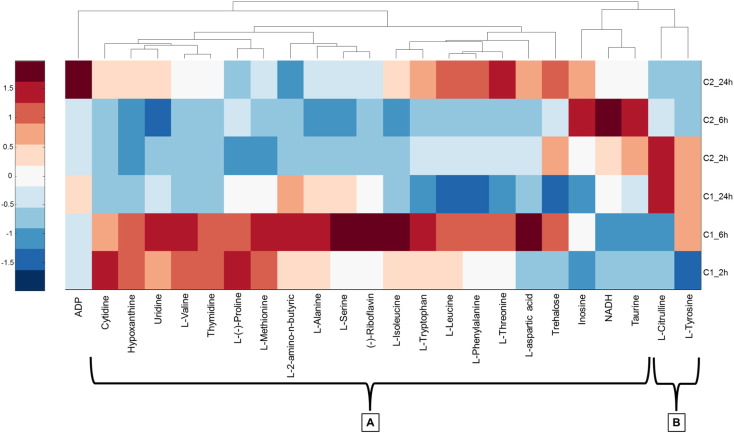
Heat map of propranolol exposure representing the fold change of targeted metabolites in each exposure subgroup (*C*_1_, *C*_2_) relative to controls. All data have been auto scaled by column and dendrograms represent hierarchical analysis for clustering (A & B) of metabolite responses.

Exposure to propranolol produced significant changes (based on two-way ANOVA, *p* < 0.05 analysis) in 8 out of 15 AAs (AABA, cytidine, l-methionine, l-proline, l-serine, taurine, l-tryptophan and l-valine), uridine (nucleoside), ADP (nucleotide) and others like hypoxanthine, riboflavin and thymidine (Table 2 and Fig. S6). Moreover, the exposure time affected 71% of the metabolites studied. l-alanine, l-aspartic acid, cytidine, l-isoleucine, l-leucine, l-methionine, l-phenylalanine, l-(−)-proline, l-threonine, l-tryptophan, l-tyrosine, l-valine, inosine, uridine, trehalose, hypoxanthine and thymidine displayed significant changes (*p* values < 0.05) across the 24 h experiments, see Table 2. Exposures at *C*_1_, a concentration below LC_10_, showed significant changes in the concentrations of the metabolites with respect to controls. For example, AABA increased its concentration by more than two-fold (Fig. S6). In other metabolites, decreasing metabolite concentrations were observed such as taurine, an essential amino acid for cardiovascular function and the central nervous system, or inosine, a nucleoside formed when hypoxanthine is attached to a ribose ring. Table S2 shows the concentrations of the metabolites determined from all exposures. At the higher exposure concentration, 153 mg L^− 1^ (*C*_2_), similar metabolite concentration changes occurred for AABA, taurine and inosine. Additionally, hypoxanthine and thymidine concentrations were different compared to controls (Fig. S6). In the samples exposed at *C*_1_, all the metabolites in Cluster A (Fig. 4) had fold decreases along the 24-h exposure time. However, l-citrulline and l-tyrosine (Cluster B, Fig. 4) and ADP had fold increases at 24-h. Finally, in the samples at *C*_2_, the general trend in Cluster A plus ADP is that the metabolites had fold increases along 24-h, with the exception of NADH and taurine. Moreover, the metabolites in Cluster B of the Fig. 4 (l-citrulline and l-tyrosine) also decreased their fold changes at 24-h exposure time.

### 3.3 Metabolic pathways potentially affected by selected single pharmaceutical exposures

Little or no reported metabolomics or pathway-based analysis data exists for *G. pulex* in the literature to our knowledge. However, changes in its metabolic profile following exposure to pharmaceuticals may result in processes that are suggestive of either metabolic (i.e. detoxification) or toxic responses. Firstly, and to support this, the internal concentrations were determined for the two compounds nimesulide and propranolol at the *C*_1_ concentration at 24-h time interval. Triclosan was detected, but was unfortunately not quantifiable due to poor standard addition linearity (and similarly poor method performance as a whole). The *C*_1_ propranolol dose resulted in measured concentrations up to 4.9 ± 0.3 mg g^− 1^ (dry weight) and nimesulide reached a mean concentration of 12.2 ± 4.1 μg g^− 1^ which are both significantly higher concentrations than the estimated dose required for therapeutic effects in humans. When comparing to environmental occurrence concentrations, these compounds did not exceed 36 ng g^− 1^ in *G. pulex* (Miller et al., 2015) and other studies in aquatic invertebrates often report concentrations of < 200 ng g^− 1^ (Dodder et al., 2014, Huerta et al., 2015). However, and although the concentrations determined here are unlikely to be seen in the environment, measurement using such analytical methods enables interpretation of metabolic responses and potentially metabolic pathways indicative of adverse effects in the future for risk assessment purposes. The different responses may be elicited through numerous complex biochemical pathways such as nucleic acid expression, protein synthesis, enzymatic processes and signaling cascades. Thus, identifying and quantifying metabolite change is a useful starting point perhaps to direct metabolomics and extended pathway-based research in the future. The advantage of operating HRMS mass analysers in full-scan mode (as in the present study) is that such further qualitative meta-analysis of the data is still possible using untargeted and/or chemometric approaches at a later time (Farrés et al., 2014). The unbiased nature of non-target metabolomics would also allow the interpretation of metabolic responses in relation to known mode-of-action pathways for pharmaceuticals. Nonetheless, this was beyond the scope of this work and future work will be pursued in this direction. A general observation was that control levels of metabolites showed relatively large scatter in specific cases. During the study the organisms were collected from the same site and were of similar size, but the variances in metabolite profiles of the controls are likely to have resulted from differences between individuals such as their age, moult cycle stage and gender. Furthermore, acclimatisation of mussel populations in laboratory conditions has also been demonstrated to lead to increased metabolic variability (Hines et al., 2007).

#### 3.3.1 Protein synthesis

Exposure to all three pharmaceuticals resulted in an increase in thymidine and inosine concentrations when compared to controls. Amongst other potential reasons, their increase in concentration could be related to increases in protein synthesis as they are associated with tRNA. Cytidine concentrations varied between exposure concentrations and time points for all compounds. Triclosan had elevated cytidine concentrations at both 2 and 6-h time intervals relative to controls in the *C*_1_ exposure. The same effect was not observed in *C*_2_, often remaining close to the control levels except at the 6-h time interval. Cytidine in the nimesulide exposure was upregulated relative to controls at *C*_1_, whereas *C*_2_ showed a steady increase in cytidine concentrations over the 24-h exposure period. For propranolol, cytidine concentrations remained below controls at *C*_2_ and were initially upregulated at *C*_1_ before decreasing below control levels at 24 h. Uridine showed no obvious differences between controls for either exposure concentration in propranolol. Uridine was upregulated in the nimesulide *C*_2_ exposure by 24-h whereas in the *C*_1_ remained higher than controls at all time points. Triclosan showed increased uridine concentrations in both exposures at the 2 and 6-h intervals when compared with control levels, the *C*_2_ uridine concentrations returned to the control level at 24-h. As uridine is absent from DNA and only present in RNA, it is plausible that together these four nucleosides are generally indicative of upregulation of protein synthesis which is potentially induced by all three pharmaceuticals. The upregulation could be considered as a general metabolic response to such xenobiotic exposure, for example, via the production of P450 enzymes (Marionnet et al., 1997, Ortiz-Delgado et al., 2008). Triclosan has also been shown to increase the P450 content of rat liver microsomes (Kanetoshi et al., 1992, Liang et al., 2013).

#### 3.3.2 Xenometabolic pathways

The internal concentrations reached in *G. pulex* exceed the human therapeutic doses and thus are likely to induce xenometabolism by means of enzymes associated with phase I and II metabolism. It is possible that the increased content of uridine is also related to phase II metabolic processes. Uridine, when converted to a triphosphate nucleotide (UTP), is involved in the biosynthesis of uridine diphosphate glucose (UDPG) which serves as a precursor of uridine glucuronic acid, the primary substrate for phase II glucuronidation reactions catalysed by uridine 5′-diphospho-glucuronosyltransferase (UGT). Major metabolites associated with these three pharmaceuticals are glucuronide conjugates, which support this argument (Macpherson et al., 2013, Walle et al., 1985, Wu et al., 2010). Riboflavin also showed statistically significant 2–3 fold increases in concentration relative to control concentrations. This metabolite is essential for xenometabolic processes as it forms part of flavin adenine nucleotide (FAD) and flavin mononucleotide (FMN), which are essential cofactors for redox reactions involving P450 enzymes and flavin-containing monooxygenases (FMOs). These cofactors are also required for regeneration of reduced glutathione (GSH) from its oxidized form glutathione disulfide (GSSG).

At *C*_1_ and *C*_2_ nimesulide exposures, AABA showed a significant reduction in concentration and also decreased over the course of the experiment. This metabolite is a precursor to ophthalmic acid, which is associated with oxidative stress and the induction of ophthalmic acid pathways is shown when glutathione levels are reduced (Soga et al., 2006). As nimesulide has been shown to reduce GSH levels as well as induce oxidative stress, it is suggestive that the low levels of AABA are a result of its conversion to ophthalmic acid, but this requires confirmation (Chatterjee et al., 2006, Mingatto et al., 2002, Singh et al., 2010). It has been suggested that the physiological significance in the production of AABA is that it is a cofactor in the transport of glucuronide metabolites in the multi-drug resistance protein 1 (MRP-1) and thus required for elimination of xenobiotics (Soga et al., 2006). Lastly, perturbations in methionine concentrations may also be indicative of xenometabolic pathways. The concentrations of methionine in all exposures were elevated relative to control with the exception of the 2 h *C*_2_ sampling point in the propranolol exposure. Methionine acts as a precursor to S-adenosyl methionine (SAM) that is involved in methylation reactions for xenobiotic metabolism. Triclosan, for example, undergoes methylation during metabolism to methyltriclosan and therefore may explain its elevated concentrations in both triclosan exposures (Wu et al., 2010). Finally, this compound also serves as a precursor to GSH biosynthesis and therefore may be elevated even when xenobiotics are not methylated during detoxification.

#### 3.3.3 Signaling cascade

Tryptophan was significantly expressed at increased concentrations in all three pharmaceutical exposures at both *C*_1_ and C_2_. This AA acts as a precursor to serotonin that is often released as a stress response. It is possible that the exposure to these pharmaceuticals induced a stress response. Indeed, *C*_2_ is set at the LOAEL with mortality as the adverse effect, which upregulated the synthesis of tryptophan for which serotonin synthesis is dependent (Joseph and Kennett, 1983). Xenobiotics have been previously shown to induce the release of serotonin in rats (Yokogoshi, 1989). However, it is also possible that tryptophan is produced in response to reactive oxygen and nitrogen species (ROS/RNS) produced by the metabolism of the pharmaceuticals (Peyrot and Ducrocq, 2008). Triclosan displayed the highest concentrations of tryptophan reaching up to 142 μg g^− 1^ when exposed at *C*_2_. The higher toxicity of this compound may lead to added stress in comparison to propranolol and nimesulide. The amino acid proline also showed statistically significant changes during the three exposures. In particular, the *C*_1_ exposures showed a decrease in proline over time until they approximately reached control levels at the 24-h time interval. The same trend is not observed in the C_2_ exposures and is more variable when compared to the controls. Proline has been shown in previous studies to be important during stress responses as this amino acid has roles in preventing oxidative stress, maintenance of osmoregulation, energy production, and many other biological functions in plants and amphipods (Maity et al., 2012, Choudhary et al., 2007).

## 4 Conclusions

A comprehensive optimisation of a targeted multi-residue method for the quantitative determination of 29 metabolites from different biochemical classes using LC-HRMS was performed. The acute toxicity (LC_50_) was also estimated for 26 pharmaceuticals and revealed that triclosan was the most toxic compound to *G. pulex*. However, the reliance on acute toxicity data such as LC_50_ for pharmaceutical risk assessment may ultimately not be realistic as these were at least one–two orders of magnitudes higher than concentrations typically found in the aquatic environment. Exposures performed at the NOAEL and LOAEL with three pharmaceuticals across the range of measured toxicity resulted in quantifiable changes in the metabolome in *G. pulex*. Measured internal concentrations of nimesulide and propranolol were far higher than the daily recommended doses for therapeutic effects in humans. Alterations in metabolite concentrations were observed, which could be involved in several different pathways relating to protein synthesis, oxidative stress and signaling cascades. However, further efforts are required to fully characterise and understand the effects these contaminants have on any specific pathway. In greater knowledge using such analytical methods for quantitative determinations of endogenous metabolites in aquatic organisms can now be acquired. In addition, the use of full-scan HRMS detection enables non-target meta-analysis to be performed in the future using chemometric tools that could identify biomarkers of exposure and effect for use in the environmental risk assessment of pharmaceuticals.

## Role of funding source

Funding bodies played no role in the design of the study or decision to publish.

## Conflict of interest

The authors declare no financial conflict of interest.

## Figures and Tables

**Table 1:** Target analytes for metabolic profiling. Kegg number, molecular formula, molecular weight (Mw) and mass spectral characterization (ordered by metabolic group) by LC-HRMS. Instrumental method performance. F: slope; R^2^: regression coefficient; IDL: instrumental detection limit; RSD: relative standard deviation; MDL: method detection limit.

Target compounds	KEGG number	Formula	Mw	Exact mass	[M-H]^−^	Linearity (ng μL^− 1^)	Calibration type	F	R^2^	IDL (ng)	Intra-day precision (5 ng μL^− 1^)	Inter-day precision (5 ng μL^− 1^)	% Recovery ± RSD, *n* = 3	MDL (ng g^− 1^)
l-2-Amino-*n*-butyric acid (AABA)	C00334	C_4_H_9_NO_2_	103.06	103.0633	102.0560	0.05–15	External	1e6	0.993	0.06	9	2	107 ± 14	13.9
l-Alanine	C00041	C_3_H_7_NO_2_	89.05	89.0477	88.0404	0.05–15	External	1e6	0.998	0.03	8	8	92 ± 9	0.99
l-Aspartic acid	C00049	C_4_H_7_NO_4_	133.04	133.0375	132.0302	0.05–15	External	4e5	0.995	0.20	4	14	87 ± 11	22.7
l-Citrulline	C00327	C_6_H_13_N_3_O_3_	175.09	175.0957	174.0884	0.1–15	External	1e6	0.999	0.15	2	6	87 ± 1	23.9
L-Isoleucine	C00407	C_6_H_13_NO_2_	131.09	131.0946	130.0873	0.1–15	Internal	4.5	0.998	0.03	12	6	42 ± 8	0.28
l-Leucine	C00123	C_6_H_13_NO_2_	131.09	131.0946	130.0873	0.1–15	Internal	1.6	0.994	0.02	13	9	99 ± 1	0.47
l-Methionine	C00073	C_5_H_11_NO_2_S	149.05	149.0510	148.0437	0.05–15	Internal	21	0.997	0.02	5	6	67 ± 14	2.66
L-Ornithine hydrochloride	C00077	C_5_H_12_N_2_O_2_	132.10	132.0899	131.0826	0.05–15	Internal	0.83	0.994	0.01	4	8	109 ± 6	3.51
l-Phenylalanine	C00079	C_9_H_11_NO_2_	165.08	165.0790	164.0717	0.1–15	Internal	1.6	0.996	0.02	14	14	97 ± 4	0.42
l-(−)-Proline	C00148	C_5_H_9_NO_2_	115.06	115.0633	114.0560	0.05–15	Internal	1.2	0.997	0.01	5	11	87 ± 8	1.13
l-Serine	C00065	C_3_H_7_NO_3_	105.04	105.0426	104.0353	0.05–15	Internal	1.1	0.996	0.01	13	13	88 ± 14	9.50
l-Threonine	C00188	C_4_H_9_NO_3_	119.05	119.0582	118.0509	0.1–15	Internal	2.0	0.999	0.2	13	7	103 ± 11	3.59
l-Tryptophan	C00078	C_11_H_12_N_2_O_2_	204.09	204.0899	203.0826	0.1–15	External	7e6	0.998	0.02	8	6	102 ± 5	1.35
l-Tyrosine	C00082	C_9_H_11_NO_3_	181.07	181.0739	180.0666	0.1–15	External	2.1	0.996	0.03	7	12	80 ± 8	0.35
l-Valine	C00183	C_5_H_11_NO_2_	117.08	117.0790	116.0717	0.05–15	Internal	1.3	0.991	0.05	12	13	91 ± 6	0.80
Cytidine	C00475	C_9_H_13_N_3_O_5_	243.08	243.0855	242.0777	0.05–15	External	3e6	0.999	0.01	8	1	110 ± 9	1.94
Inosine	C00294	C_10_H_12_N_4_O_5_	268.09	268.0808	267.0734	0.01–15	Internal	1.01	0.995	0.002	1	14	108 ± 8	0.04
Thymidine	C00214	C_10_H_14_N_2_O_5_	242.20	242.0903	241.0829	0.05–15	Internal	0.7	0.991	0.01	7	5	99 ± 7	0.50
Uridine	C00299	C_9_H_12_N_2_O_6_	244.07	244.0695	243.0622	0.05–15	External	2e6	0.997	0.03	7	5	138 ± 2	0.33
ADP	C00008	C_10_H_15_N_5_O_10_P_2_	427.03	427.0294	426.0221	3–15	External	7e5	0.993	0.15	9	11	25 ± 11	162
NADH	C00004	C_21_H_27_N_7_O_14_P_2_	663.07	663.1091	662.1018	0.1–15	External	1e6	0.990	0.06	2	19	162 ± 10	7.80
Trehalose	C01083	C_12_H_22_O_11_	342.12	342.1162	341.1089	0.05–15	External	2e6	0.996	0.01	10	0.2	81 ± 7	1.03
Creatine	C00300	C_4_H_9_N_3_O_2_	131.07	131.0695	130.0622	0.1–15	External	3e5	0.991	0.11	3	0.5	107 ± 2	37.3
Phthalic acid	C01606	C_8_H_6_O_4_	166.14	166.0266	165.0193	0.1–15	External	1e6	0.997	0.01	6	7	102 ± 2	13.6
Taurine	C00245	C_2_H_7_NO_3_S	125.01	125.0147	124.0073	0.05–15	Internal	2.03	0.992	0.03	8	14	92 ± 20	0.23
1,7-Dimethylxanthine	C13747	C_7_H_8_N_4_O_2_	180.20	180.0647	179.0574	0.05–15	Internal	1.19	0.997	0.01	10	13	72 ± 4	2.33
Hypoxanthine	C00262	C_5_H_4_N_4_O	136.04	136.0385	135.0312	0.01–15	External	8e6	0.991	0.004	4	9	102 ± 7	0.47
Pyridoxine	C00314	C_8_H_11_NO_3_	169.20	169.0739	168.0666	0.05–15	External	3e6	0.94	0.07	6	0.1	105 ± 10	21.6
(−)-Riboflavin	C00255	C_17_H_20_N_4_O_6_	376.37	376.1383	375.1310	0.1–15	Internal	0.9	0.996	0.04	12	9	89 ± 7	6.50
^13^C,^15^N-Isoleucine^a^	–	C_6_H_13_NO_2_	138.12	138.1115	137.1041	–	–	–	–	–	–	–	–	–
^13^C,^15^N-Leucine^a^	–	C_6_H_13_NO_2_	138.12	138.1115	137.1041	–	–	–	–	–	–	–	–	–
^13^C,^15^N-Methionine^a^	–	C_5_H_11_NO_2_S	155.17	155.0646	154.0573	–	–	–	–	–	–	–	–	–
^13^C,^15^N-Phenylalanine^a^	–	C_9_H_11_NO_2_	175.12	175.1057	174.0984	–	–	–	–	–	–	–	–	–
^13^C,^15^N-Proline^a^	–	C_5_H_9_NO_2_	121.09	121.0769	120.0695	–	–	–	–	–	–	–	–	–
^13^C,^15^N-Serine^a^	–	C_3_H_7_NO_3_	109.08	109.0495	108.0422	–	–	–	–	–	–	–	–	–
^13^C,^15^N-Threonine^a^	–	C_4_H_9_NO_3_	124.08	124.0685	123.0611	–	–	–	–	–	–	–	–	–
^13^C,^15^N-Valine^a^	–	C_5_H_11_NO_2_	123.10	123.0925	122.0852	–	–	–	–	–	–	–	–	–

a Internal standards used to quantify (Algal AA mixture).

**Table 2:** Two-way ANOVA indicating *p* values for changes in metabolite concentrations based on time and dose factor. The significant changes are in bold where *p* < 0.05.

	*p*-Value								
	Triclosan			Nimesulide			Propanolol		
Metabolite	Dose factor^a^	Time factor^b^	Interaction dose × time	Dose factor^a^	Time factor^b^	Interaction Dose × Time	Dose factor^a^	Time factor^b^	Interaction dose × time
AABA	0.49	0.23	0.91	**–**	**3e − 4**	**0.003**	**–**	0.24	0.32
l-Alanine	**–**	0.19	0.15	0.53	**–**	**6e − 4**	**–**	**0.001**	**0.03**
l-Aspartic acid	0.17	0.37	0.25	**2e − 4**	**0.005**	**0.004**	0.28	**0.04**	0.44
Cytidine	**0.001**	0.08	**0.02**	**–**	**0.02**	**–**	**5e − 6**	**1e − 5**	**1e − 6**
l-Citrulline	**0.01**	0.81	0.95	**4e − 6**	**2e − 5**	**8e − 6**	0.53	0.29	0.07
l-Isoleucine	**–**	**9e − 4**	0.12	**–**	0.29	**–**	0.06	**3e − 4**	**0.003**
l-Leucine	**0.01**	0.47	0.73	**–**	**0.003**	**–**	0.25	**–**	**–**
l-Methionine	**–**	0.18	**0.003**	**–**	0.15	**1e − 4**	**0.003**	**0.005**	**0.007**

l-Ornithine hydrochloride	Not detected in *Gammarus pulex* organism								
l-Phenylalanine	**–**	**0.001**	**0.001**	**–**	0.53	**0.0001**	0.69	**–**	**0.001**
l-(−)-Proline	**–**	**0.001**	**0.01**	**4e − 7**	**6e − 13**	**8e − 9**	**–**	**–**	**2e − 4**
l-Serine	0.21	0.05	0.23	**0.04**	**0.02**	**0.03**	**–**	0.73	**0.01**
Taurine	**–**	**0.002**	0.10	**–**	0.58	**0.002**	**0.03**	0.05	0.75
l-Threonine	**–**	**0.03**	0.07	**–**	0.07	**0.001**	0.34	**–**	**0.001**
l-Tryptophan	**–**	0.28	0.53	**–**	0.08	**–**	**0.0012**	**–**	**–**
l-Tyrosine	**–**	**0.01**	0.40	**0.003**	0.97	0.12	0.70	**0.01**	0.27
l-Valine	**–**	**0.01**	0.05	**–**	0.42	**–**	**5e − 4**	**2e − 4**	**–**
Inosine	**–**	**0.01**	0.11	**–**	0.17	**0.03**	0.12	**0.002**	0.27
Uridine	**8e − 4**	0.45	0.26	**–**	0.98	**–**	**0.002**	**6e − 4**	**0.009**
ADP	0.21	0.25	0.74	**0.04**	0.14	**0.01**	**0.02**	0.87	0.64
NADH	0.05	0.41	0.31	0.49	0.72	0.12	0.52	0.58	0.83
Trehalose	**0.03**	0.11	0.08	0.07	**–**	**–**	0.21	**–**	**–**

Creatine	Not detected in *Gammarus pulex* organism								
1,7-dimethylxanthine	Not detected in *Gammarus pulex* organism								
Hypoxanthine	**–**	0.08	0.07	**–**	0.14	**–**	**1e − 6**	**4e − 5**	**8e − 7**

Phtalic acid	Not detected in *Gammarus pulex* organism								
Pyridoxine (vitamin B6)	Not detected in *Gammarus pulex* organism								
(−)-Riboflavin	**0.01**	0.49	0.36	**–**	0.46	**0.002**	**3e − 4**	0.69	**0.008**
Thymidine	**–**	**0.01**	0.12	**–**	**7e − 4**	**–**	**4e − 4**	**0.009**	**2e − 4**

a Dose factor corresponds at the two different concentrations used for each pharmaceutical.

b Time factor corresponds at the different times studied (2, 6 and 24 h).

## Appendix A Supplementary data

Supplementary material Image 2
