# Supplementary material for: Targeted metabolomics of Gammarus pulex following controlled exposures to selected pharmaceuticals in water
Source: Sci Total Environ. 2016 Aug 15;562:777–88. doi: 10.1016/j.scitotenv.2016.03.181 (PMC4912218; doi:10.1016/j.scitotenv.2016.03.181)
Supplement: Supplementary file 1 — Supplementary material [file mmc1.docx]

**Supplementary material**

**Targeted metabolomics of *Gammarus pulex* following controlled exposures to selected pharmaceuticals in water**

Cristian Gómez-Canela^a,b*^, Thomas H. Miller^b^, Nicolas R. Bury^c^, Romà Tauler^a^ and Leon P. Barron^b^

*^a^Department of Environmental Chemistry, IDAEA-CSIC, Jordi Girona 18-26, 08034 Barcelona, Catalonia, Spain.*

*^b^Analytical & Environmental Sciences Division, Faculty of Life Sciences and Medicine, King's College London, 150 Stamford Street, London SE1 9NH, UK.*

*^c^Diabetes and Nutritional Sciences, Faculty of Life Sciences and Medicine, King’s College London, 150 Stamford Street, London SE1 9NH, UK.*

*Corresponding author

Email: cristian.gomez@cid.csic.es (C. Gómez-Canela)

Tel: +34 93 400 61 00

Fax: +34 93 204 59 04

**Table S1.** Pharmaceuticals studied herein and some relevant physicochemical properties (ordered by their ATC code system).

| **Drug** | **ATC code** | **ATC group** | **CAS number** | **Molecular formula** | **Mw**  **(g mol^-1^)** | **Water solubility**  **(mg L^-1^)^a^** | **log P^b^** | **pKa^b^** |
| --- | --- | --- | --- | --- | --- | --- | --- | --- |
| **Ranitidine** | A02BA02 | Drugs for peptic ulcer and gastro-oesophageal reflux disease (GORD) | 66357-35-5 | C_13_H_22_N_4_O_3_S | 314.86 | 1800 | 0.2 | 8.8 |
| **Warfarin** | B01AA03 | Antithrombotic agents | 81-81-2 | C_19_H_16_O_4_ | 308.32 | 17 | 2.7 | 4.8 |
| **Furosemide** | C03CA01 | High-Ceiling diuretics | 54-31-9 | C_12_H_11_ClN_2_O_5_S | 330.74 | 73.1 | 0.43 | 3.8 |
| **(±)-Metoprolol** | C07AB02 | Beta blockers | 56392-17-7 | C_34_H_56_N_2_O_12_ | 684.82 | 16900 | 1.88 | 9.7 |
| **Atenolol** | C07AA03 |  | 56715-13-0 | C_14_H_22_N_2_O_3_ | 266.34 | 13300 | 0.23 | 9.6 |
| **Propanolol** | C07AA05 |  | 525-66-6 | C_16_H_21_NO_2_·HCl | 295.80 | 50000 | 3.65 | 9.4 |
| **Clofibric acid** | C10AB01 | Lipid modifying agents, plain | 882-09-7 | C_10_H_11_ClO_3_ | 214.64 | 582.5 | 2.72 | 3.0 |
| **Bezafibrate** | C10AB02 |  | 41859-67-0 | C_19_H_20_ClNO_4_ | 361.82 | Insoluble | -0.4 | 3.6 |
| **Gemfibrozil** | C10AB04 |  | 25812-30-0 | C_15_H_22_O_3_ | 250.33 | <1000 | 2.8 | 4.7 |
| **Triclosan** | D08AE04 | Antiseptics and disinfectants | 3380-34-5 | C_12_H_7_Cl_3_O_2_ | 289.54 | 12000 (20 ºC) | 5 | 8.1 |
| **Triclocarban** | - |  | 101-20-2 | C_13_H_9_Cl_3_N_2_O | 315.58 | 10 (26 ºC) | 4.76 | 12.7 |
| **Trimethoprin** | J01EA01 | Sulfonamides and trimethoprim | 738-70-5 | C_14_H_18_N_4_O_3_ | 290.3 | 400 | 0.91 | 7.4 |
| **Sulfamethoxazole** | J01EC01 |  | 723-46-6 | C_10_H_11_N_3_O_3_S | 253.28 | 610 (37 ºC) | 0.89 | 6.16 |
| **Tamoxifen** | L02BA01 | Hormone antagonists and related agents | 10540-29-1 | C_26_H_29_NO | 371.51 | 0.167 | 7.1 | 8.76 |
| **Indomethacin** | M01AB01 | Antiinflammatory and antirheumatic products | 53-86-1 | C_19_H_16_ClNO_4_ | 357.79 | 0.937 | 3.1 | 4.5 |
| **Sodium diclofenac** | M01AB05 |  | 15307-79-6 | C_14_H_10_Cl_2_NO_2_·Na | 318.13 | 50000 | 4.4 | 4.22 |
| **Ketoprofen** | M01AE03 |  | 22071-15-4 | C_16_H_14_O_3_ | 254.28 | <1000 | 0.97 | 4.6 |
| **Flurbiprofen** | M01AE09 |  | 5104-49-4 | C_15_H_13_FO_2_ | 244.3 | 8 | 4.2 | 4.2 |
| **Ibuprofen** | M01AE51 |  | 15687-27-1 | C_13_H_17_O_2_·Na | 228.3 | 100000 | 3.5 | 4.4 |
| **Meclofenamic acid** | M01AG04 |  | 1185072-18-7 | C_14_H_10_Cl_2_NO_2_·Na | 318.13 | 30 | - | 3.76 |
| **Nimesulide** | M01AX17 |  | 51803-78-2 | C_13_H_12_N_2_O_5_S | 308.30 | 10 | 2.39 | 6.5 |
| **Carbamazepine** | N03AF01 | Antiepileptics | 298-46-4 | C_15_H_12_N_2_O | 236.27 | 112 | 2.67 | 14 |
| **Imipramine** | N06AA02 | Antidepressants | 50-49-7 | C_19_H_24_N_2_·HCl | 316.87 | 50000 | 4.80 | 9.4 |
| **Amitriptyline** | N06AA09 |  | 549-18-8 | C_20_H_23_N | 277.4 | 9.71 (24 ºC) | 4.92 | 9.76 |
| **Nortriptyline** | N06AA10 |  | 894-71-3 | C_19_H_21_N | 263.37 | 0.874 | 4.65 | 10.47 |
| **Salicylic acid** | S01BC08 | Antiinflammatory agents | 69-72-7 | C_7_H_6_O_3_ | 138.12 | 1800 | 2.26 | 2.97 |

^a^Solubility values were extracted from Drugbank (http://www.drugbank.ca/drugs/). ^b^ log P and pK_a_ values were extracted from the Estimation Program Interface (EPI) suite program (Environmental Protection Agency).

| **Table S2.** Concentrations of target metabolites (µg g^−1^) ± standard deviation (sd) in *G. pulex* following the exposure trial using three selected pharmaceuticals: propranolol, triclosan and nimesulide. | | | | | | | | | | |
| --- | --- | --- | --- | --- | --- | --- | --- | --- | --- | --- |
|  |  | **Propanolol (µg g^-1^) ± sd** | | | **Triclosan (µg g^-1^) ± sd** | | | **Nimesulide (µg g^-1^) ± sd** | | |
| **Metabolite** | **Exposition time** | **Control** | **C1= 100 mg/L** | **C2= 153 mg/L** | **Control** | **C1= 0.1 mg/L** | **C2= 0.3 mg/L** | **Control** | **C1= 0.5 mg/L** | **C2= 1.4 mg/L** |
| L-2-amino-n-butyric | **2h** | 7.6±4 | 18.9±4 | 18.1±3 | 17.7±1 | 15.1±3 | 17.0±2 | 22.1±3 | 8.7±0.4 | 7.5±1 |
|  | **6h** | 6.1±2 | 27.1±3 | 21.4±0.7 | 22.7±4 | 21.1±4 | 19.8±2 | 22.1±8 | 5.9±0.5 | 7.1±2 |
|  | **24h** | 8.2±3 | 23.9±0.5 | 18.2±0.4 | 22.5±6 | 16.8±5 | 21.5±2 | 6.1±1 | 3.8±0.9 | 6.2±1 |
| L-Alanine | **2h** | 641±80 | 822±131 | 656±44 | 508±2 | 999±2 | 827±10 | 488±13 | 366±9 | 357±10 |
|  | **6h** | 486±28 | 799±70 | 541±11 | 531±25 | 1119±48 | 784±31 | 326±15 | 354±7 | 276±0.3 |
|  | **24h** | 492±42 | 583±70 | 548±1 | 605±5 | 810±110 | 673±17 | 212±8 | 268±6 | 324±2 |
| L-aspartic acid | **2h** | 67.1±9 | 75.8±26 | 63.1±27 | 55.8±7 | 62.6±3 | 60.5±3 | 36.3±12 | 54.7±0.5 | 20.1±2 |
|  | **6h** | 47.3±17 | 55.6±14 | 69.0±16 | 59.3±4 | 43.2±2 | 36.1±6 | 38.6±6 | 81.9±11 | 25.7±4 |
|  | **24h** | 73.5±2 | 73.0±26 | 99.9±5 | 56.8±4 | 82.5±26 | 33.9±11 | 18.6±0.5 | 28.5±0.5 | 34.4±2 |
| Cytidine | **2h** | 16±10 | 54±30 | 9±3 | 16±6 | 34±22 | 15±6 | 11±3 | 28±1 | 5±0.5 |
|  | **6h** | 11±3 | 21±0.2 | 6±0.2 | 12±5 | 66±21 | 19±7 | 4±0.3 | 37±5 | 16±10 |
|  | **24h** | 20±11 | 3±5 | 6±8 | 21±1 | 19±3 | 16±3 | 2±0.3 | 16±0.5 | 24±2 |
| L-Citrulline | **2h** | 8.9±0.6 | 7.3±1 | 11.21± | 8.9±0.7 | 9.7±0.4 | 14.6±0.4 | 12.8±0.5 | 8.0±0.3 | 5.9±0.6 |
|  | **6h** | 11.8±1 | 9.7±0.2 | 10.42± | 9.2±0.8 | 9.0±0.9 | 12.3±1 | 6.9±0.7 | 7.0±0.2 | 5.5±0.3 |
|  | **24h** | 7.2±1 | 11.1±0.5 | 9.4±1 | 8.9±2 | 8.3±0.5 | 13.8±2 | 8.9±0.1 | 7.4±0.3 | 9.0±0.4 |
| L-Isoleucine | **2h** | 1129±144 | 1319±83 | 1243±32 | 734±80 | 2067±110 | 1140±38 | 857±4 | 1071±51 | 728±27 |
|  | **6h** | 811±114 | 1432±82 | 1009±50 | 1066±216 | 2685±88 | 1561±147 | 608±51 | 1252±50 | 619±13 |
|  | **24h** | 902±177 | 667±22 | 961±96 | 941±35 | 1743±224 | 1164±39 | 597±23 | 771±27 | 1190±22 |
| L-Leucine | **2h** | 452±52 | 522±15 | 446±11 | 277±27 | 443±1 | 361±17 | 228±3 | 274±10 | 231±5 |
|  | **6h** | 336±57 | 472±31 | 384±16 | 318±45 | 459±4 | 353±4 | 129±5 | 313±8 | 140±15 |
|  | **24h** | 433±31 | 241±19 | 332±7 | 318±28 | 376±13 | 299±0.7 | 183±9 | 215±3 | 258±2 |
| L-Methionine | **2h** | 2.5±0.5 | 3.5±1 | 2.5±0.3 | 2.0±0.1 | 5.4±0.6 | 3.8±0.6 | 2.9±0.6 | 5.6±0.1 | 3.5±0.3 |
|  | **6h** | 2.4±0.1 | 3.4±0.1 | 3.0±0.3 | 2.8±0.7 | 5.0±0.5 | 4.6±0.8 | 3.7±0.6 | 5.2±0.7 | 4.3±0.1 |
|  | **24h** | 1.9±0.1 | 3.5±0.1 | 3.0±0.3 | 2.6±0.6 | 7.0±0.3 | 3.1±0.9 | 2.7±0.1 | 4.2±0.2 | 5.3±0.4 |
| L-Phenylalanine | **2h** | 146±1.5 | 135±50 | 148±5 | 70±14 | 340±28 | 200±15 | 125±17 | 193±7 | 141±9 |
|  | **6h** | 105±19 | 168±7 | 118±22 | 98±10 | 417±5 | 233±5 | 128±3 | 182±7 | 126±0.8 |
|  | **24h** | 115±10 | 59±2 | 77±9 | 134±3 | 260±6 | 134±0.5 | 110±1 | 153±1 | 185±4 |
| L-(-)-Proline | **2h** | 221±22 | 387±55 | 278±11 | 238±4 | 499±11 | 313±1 | 234±3 | 293±4 | 222±1 |
|  | **6h** | 206±12 | 314±9 | 294±12 | 268±7 | 462±5 | 246±4 | 211±3 | 241±1 | 237±2 |
|  | **24h** | 206±9 | 228±7 | 241±9 | 249±9 | 293±13 | 207±3 | 174±7 | 173±3 | 205±3 |
| L-Serine | **2h** | 87.4±13 | 125±13 | 97.9±15 | 130±7 | 158±7 | 158±7 | 120±4 | 86.7±0.7 | 87.3±0.8 |
|  | **6h** | 61.6±19 | 147±10 | 109.6±8 | 165±18 | 179±13 | 168±2 | 99.8±11 | 103±3 | 87.6±0.7 |
|  | **24h** | 83.6±23 | 119±2 | 123.1±8 | 139±2 | 120±12 | 167±15 | 82.7±2 | 80.7±2 | 86.0±1 |
| Taurine | **2h** | 22781±6142 | 13010±1169 | 13014±951 | 5183±438 | 7300±168 | 7645±212 | 8651±348 | 42553±951 | 15126±1334 |
|  | **6h** | 22070±295 | 8848±167 | 14360±296 | 5313±3 | 10516±434 | 8484±60 | 8934±650 | 34433±18 | 18343±1469 |
|  | **24h** | 10222±1768 | 6790±202 | 7476±534 | 7345±857 | 12433±2122 | 8468±142 | 7838±234 | 33298±198 | 25007±549 |
| L-Threonine | **2h** | 484±39 | 526±21 | 463±9 | 313±21 | 740±6 | 530±7 | 328±18 | 767±20 | 474±10 |
|  | **6h** | 394±50 | 435±12 | 373±20 | 399±25 | 787±22 | 486±8 | 368±17 | 627±12 | 478±18 |
|  | **24h** | 395±39 | 259±5 | 359±36 | 371±18 | 600±20 | 440±11 | 321±10 | 519±3 | 542±18 |
| L-Tryptophan | **2h** | 80±9 | 96±11 | 72±5 | 43±8 | 74±20 | 142±7 | 80±12 | 90±3 | 66±4 |
|  | **6h** | 60±6 | 93±7 | 63±2 | 56±14 | 97±16 | 134±10 | 44±7 | 103±10 | 77±13 |
|  | **24h** | 61±9 | 35±2 | 44±6 | 52±12 | 72±8 | 115±8 | 32±3 | 81±14 | 93±6 |
| L-Tyrosine | **2h** | 177±22 | 160±24 | 163±10 | 152±11 | 247±8 | 216±5 | 209±0.1 | 244±6 | 226±8 |
|  | **6h** | 149±19 | 160±4 | 143±10 | 170±20 | 301±1 | 234±2 | 217±6 | 241±4 | 223±8 |
|  | **24h** | 130±15 | 151±7 | 147±19 | 181±7 | 307±4 | 224±1 | 178±7 | 240±13 | 255±6 |
| L-Valine | **2h** | 211±28 | 351±0.3 | 234±11 | 153±0.7 | 500±1 | 268±1 | 201±4 | 437±7 | 245±0.4 |
|  | **6h** | 188±35 | 285±4 | 231±16 | 195±15 | 601±9 | 299±18 | 162±0.1 | 429±0.6 | 278±2 |
|  | **24h** | 229±44 | 166±7 | 201±11 | 198±15 | 398±17 | 250±3 | 195±0.5 | 308±2 | 330±2 |
| Inosine | **2h** | 53345±3516 | 36739±1695 | 28770±7437 | 8814±1784 | 14808±1341 | 20640±179 | 20169±378 | 31192±6407 | 111987±81 |
|  | **6h** | 23237±888 | 22687±2581 | 28394±1283 | 8430±258 | 18116±26 | 23098±627 | 17304±1233 | 39476±13106 | 98822±889 |
|  | **24h** | 24884±5019 | 12815±566 | 15620±919 | 11014±587 | 18957±5084 | 36017±80 | 13982±750 | 80007±6869 | 104115±3365 |
| Uridine | **2h** | 459±68 | 471±132 | 453±22 | 440±4 | 699±21 | 506±66 | 370±18 | 496±14 | 302±5 |
|  | **6h** | 402±45 | 317±43 | 474±9 | 430±0.1 | 723±21 | 533±28 | 332±2 | 512±28 | 315±0.1 |
|  | **24h** | 440±40 | 401±23 | 371±31 | 593±39 | 644±77 | 574±15 | 256±1 | 398±1 | 500±9 |
| ADP | **2h** | 20.0±3 | 0±0 | 28.4±3 | 41.5±5 | 28.6±2 | 30.5±0.2 | 30.9±0.7 | 29.1±0.6 | 27.5±0.2 |
|  | **6h** | 19.8±1 | 0±0 | 27.7±1 | 34.7±0.3 | 18.6±3 | 28.2±0.3 | 29.0±0.7 | 27.0±0.1 | 27.9±0.9 |
|  | **24h** | 8.8±0 | 9.1±0 | 24.5±9 | 22.1±4 | 18.7±1 | 29.3±0.8 | 26.7±0.7 | 28.4±0.6 | 26.7±0.5 |
| NADH | **2h** | 10.7±1 | 8.1±2 | 13.8±1 | 11.7±1 | 7.11±0.1 | 9.3±0.2 | 8.6±0.4 | 4.9±0.3 | 7.8±0.1 |
|  | **6h** | 9.3±1 | 8.1±0.5 | 7.5±0.4 | 10.7±0.1 | 7.7±1 | 8.1±0.8 | 7.6±0.1 | 7.4±0.4 | 7.7±0.1 |
|  | **24h** | 8.6±1 | 8.3±0.5 | 8.3±0.5 | 7.1±2 | 7.7±0.1 | 9.9±0.3 | 4.9±0.2 | 7.7±0.1 | 7.8±0.1 |
| Trehalose | **2h** | 422±19 | 351±63 | 420±16 | 280±16 | 197±1 | 272±10 | 145±26 | 191±1 | 152±5 |
|  | **6h** | 290±40 | 426±25 | 348±6 | 268±3 | 259±8 | 251±4 | 150±4 | 162±6 | 201±0.4 |
|  | **24h** | 277±36 | 131±4 | 170±22 | 265±17 | 251±22 | 378±17 | 166±0.2 | 130±6 | 72±0.1 |
| Hypoxanthine | **2h** | 46±7 | 44±15 | 85±1 | 30±14 | 123±4 | 74±22 | 35±8 | 71±11 | 32±1 |
|  | **6h** | 39±6 | 26±4 | 70±0.5 | 38±13 | 154±27 | 74±18 | 22±3 | 71±9 | 34±12 |
|  | **24h** | 47±5 | 42±1 | 28±5 | 48±29 | 95±27 | 53±10 | 22±0.5 | 46±8 | 81±6 |
| (-)-Riboflavin | **2h** | 1.6±0.5 | 2.6±1 | 1.1±0.7 | 1.5±0.6 | 2.6±0.7 | 2.4±0.5 | 2.6±0.4 | 3.5±0.4 | 1.9±0.1 |
|  | **6h** | 0.5±0.1 | 3.1±0.3 | 1.5±0.5 | 1.6±1 | 3.4±3 | 2.6±0.3 | 1.7±0.3 | 4.7±0.5 | 2.3±0.5 |
|  | **24h** | 1.3±0.4 | 2.0±0.1 | 2.5±0.6 | 2.2±2 | 2.4±2 | 2.6±0.8 | 1.1±0.1 | 4.2±0.7 | 2.8±0.7 |
| Thymidine | **2h** | 669±52 | 530±2 | 2655±78 | 89±10 | 2918±603 | 639±140 | 281±82 | 2719±72 | 472±72 |
|  | **6h** | 488±7 | 535±18 | 1520±111 | 216±49 | 3697±180 | 1163±130 | 152±36 | 3042±60 | 929±250 |
|  | **24h** | 1114±74 | 437±52 | 237±33 | 128±37 | 1718±19 | 396±12 | 349±10 | 1079±27 | 1250±125 |

**Table S3.** Toxicity of 26 pharmaceuticals to *Gammarus pulex* and comparison with values reported in the literature.

|  |  |  | 24 h- LC_50_ (mg L^-1^) | |
| --- | --- | --- | --- | --- |
|  | CAS number | Range of concetrations tested (mg L^-1^) | *G. pulex*  (present study) | *T. platyurus^1^* |
| Triclosan | 3380-34-5 | 0.1-100 | 0.57 | 0.47 |
| Nimesulide | 51803-78-2 | 0.5-100 | 1.87 | - |
| Imipramine | 50-49-7 | 3-50 | 3.85 | - |
| Meclofenamic | 644-62-2 | 0.1-50 | 4.54 | - |
| Gemfibrozil | 25812-30-0 | 0.1-100 | 9.84 | - |
| Nortriptyline | 72-69-5 | 10-100 | 18.86 | - |
| Ketoprofen | 22071-15-4 | 20-100 | 22.5 | - |
| Salicylic acid | 69-72-7 | 10-100 | 29.29 | - |
| Amitriptyline | 50-48-6 | 10-100 | 41.75 | - |
| Diclofenac | 15307-86-5 | 10-100 | 42.22 | - |
| Ibuprofen | 15687-27-1 | 1-100 | 44.02 | 19.59 |
| Tamoxifen | 10540-29-1 | 0.01-100 | >0.16* | - |
| Indomethacin | 53-86-1 | 1-100 | >0.93* | 16.14 |
| Flurbiprofen | 5104-49-4 | 0.1-10 | >8* | - |
| Triclocarban | 101-20-2 | 0.05-50 | >10* | - |
| Warfarin | 81-81-2 | 0.1-20 | >17* | - |
| Furosemide | 54-31-9 | 1-75 | >73.1* | - |
| Atenolol | 29122-68-7 | 1-100 | >100 | >100 |
| Sulfamethoxazole | 723-46-6 | 1-150 | 102.79 | - |
| Carbamazepine | 298-46-4 | 1-150 | >112* | >100 |
| Propranolol | 525-66-6 | 10-200 | 165.15 | 10.31 |
| Metoprolol tartrate | 51384-51-1 | 1-150 | >150 | - |
| Trimethoprin | 738-70-5 | 1-200 | >200 | - |
| Ranitidine | 66357-35-5 | 1-250 | >250 | - |
| Clofibric acid | 882-09-7 | 1-250 | >250 | - |
| Bezafibrate | 41859-67-0 | 0.01-50 | >1* | - |

The LC_10_ values of propranolol, triclosan and nimesulide were 152.73, 0.20 and 0.65 mg L^-1^.

* LC_50_ is above solubility.

^1^ Kim, J.W., et al. Journal of Toxicological Sciences, 2009. 34(2): p. 227-232.

**
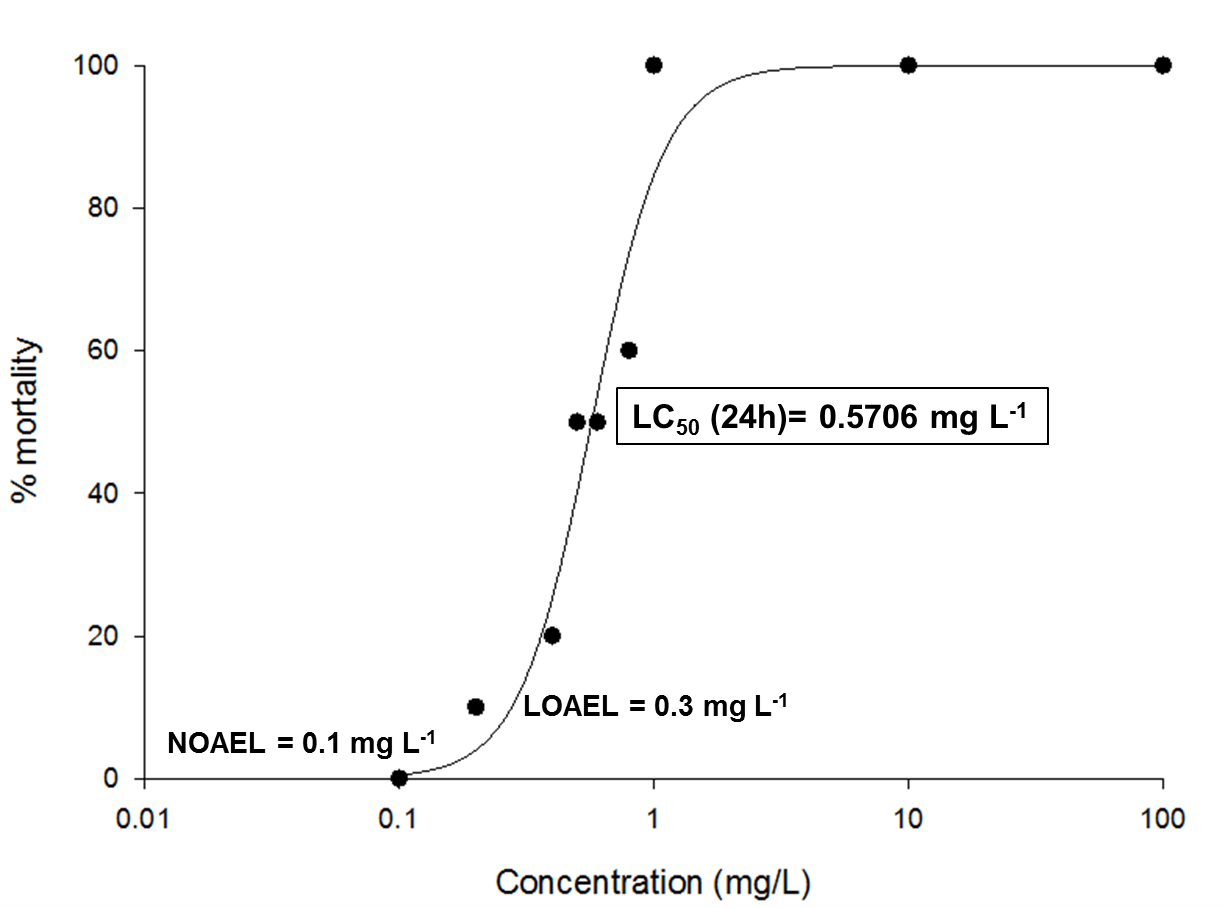
**

**Figure S1.** Triclosan dose-response curve adjusted to the Hill regression model in *G. pulex*. Estimated LC_50_ is also depicted. Each point is a single measurement.


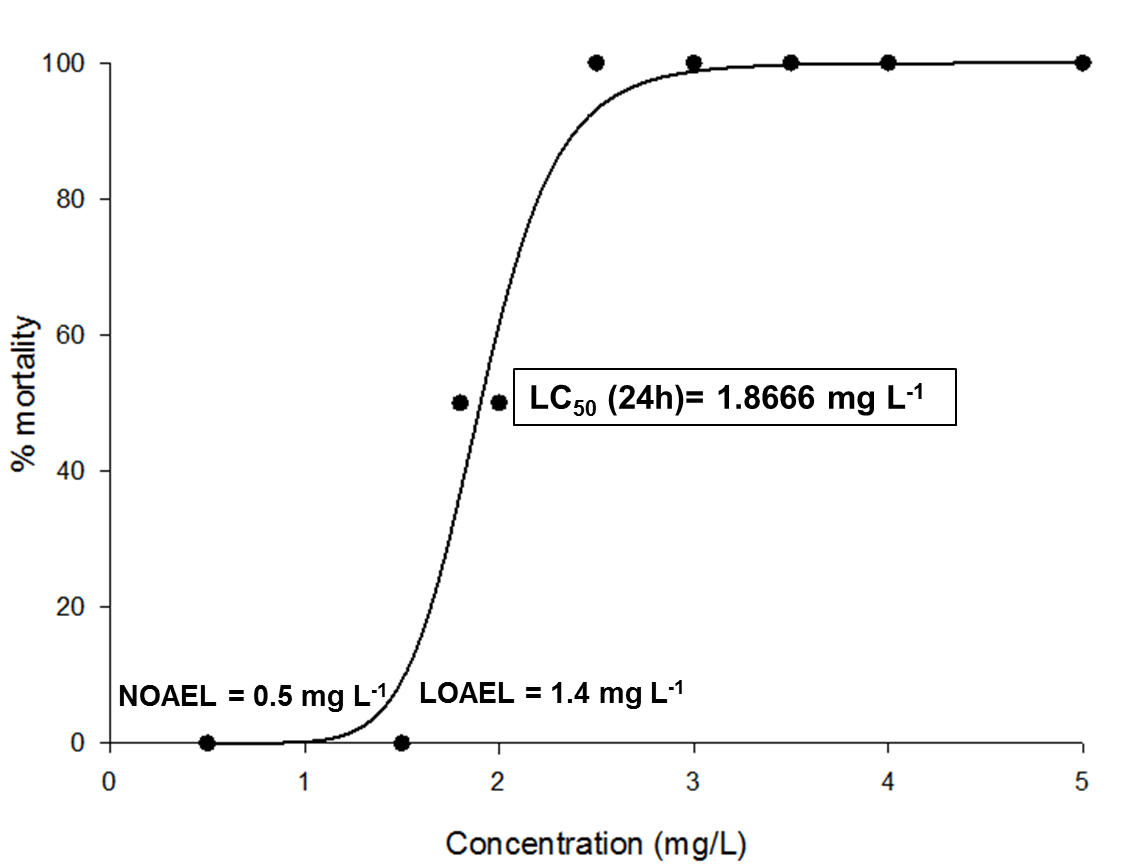


**Figure S2.** Nimesulide dose-response curve adjusted to the Hill regression model in *G. pulex*. Estimated LC_50_ is also depicted. Each point is a single measurement.

**
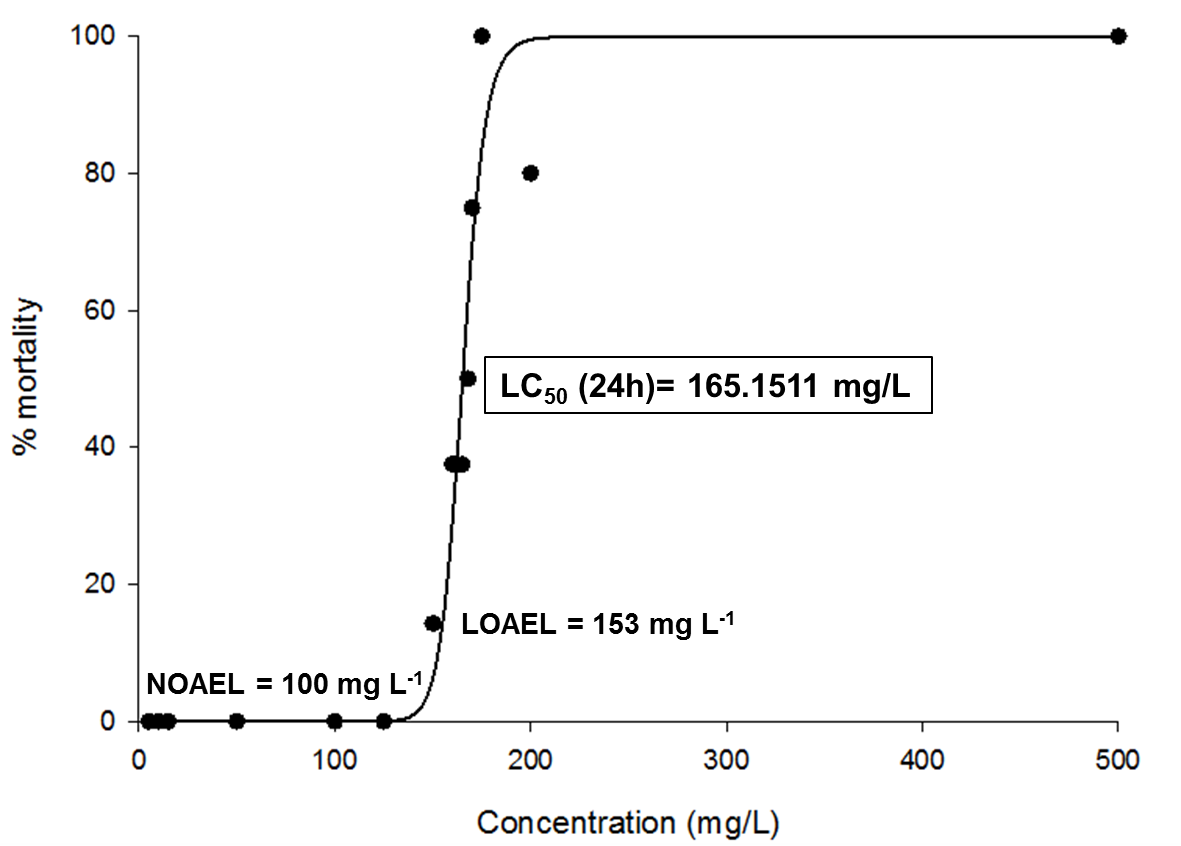
**

**Figure S3.** Propranolol dose-response curve adjusted to the Hill regression model in *G. pulex*. Estimated LC_50_ is also depicted. Each point is a single measurement.


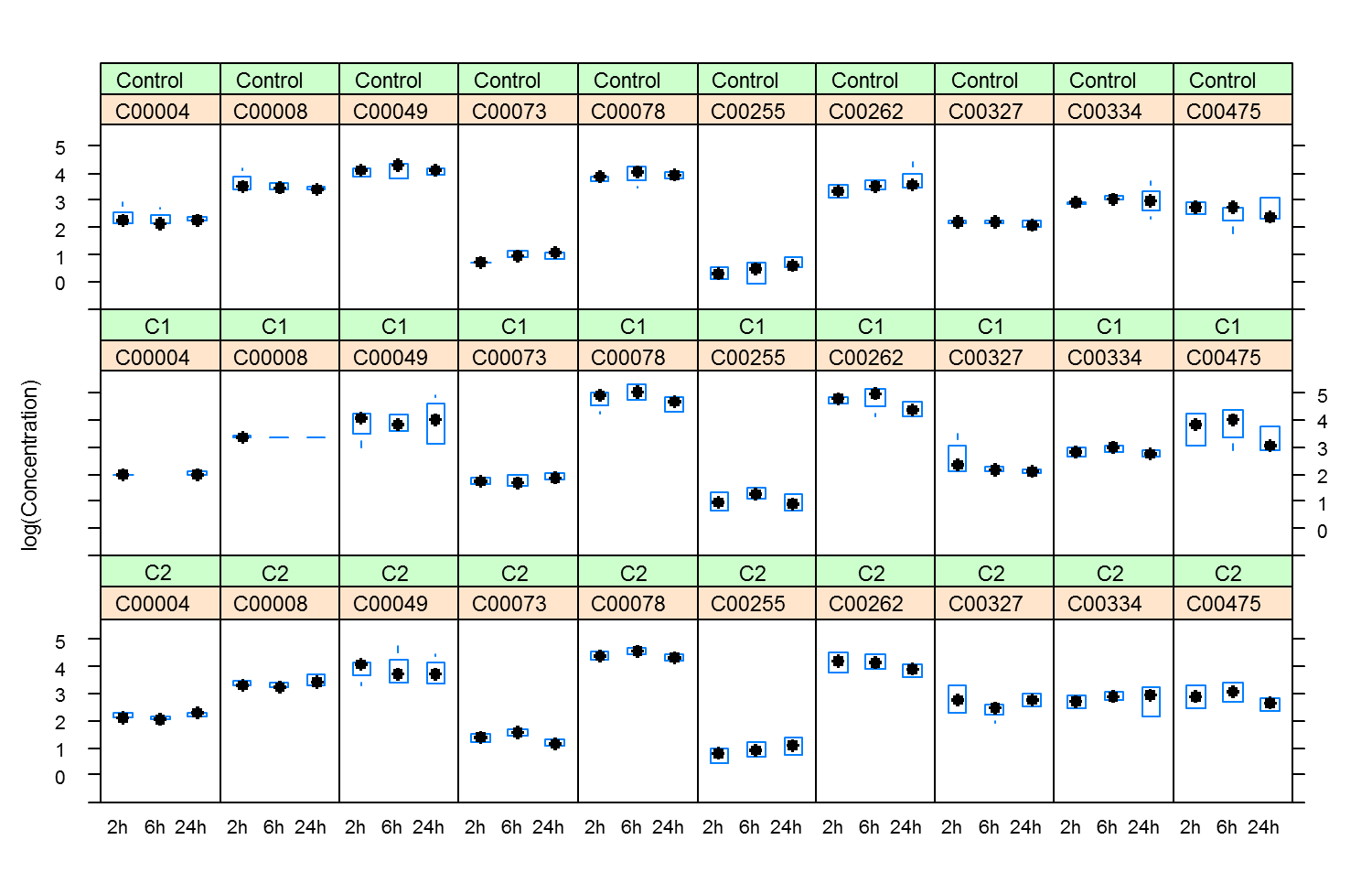


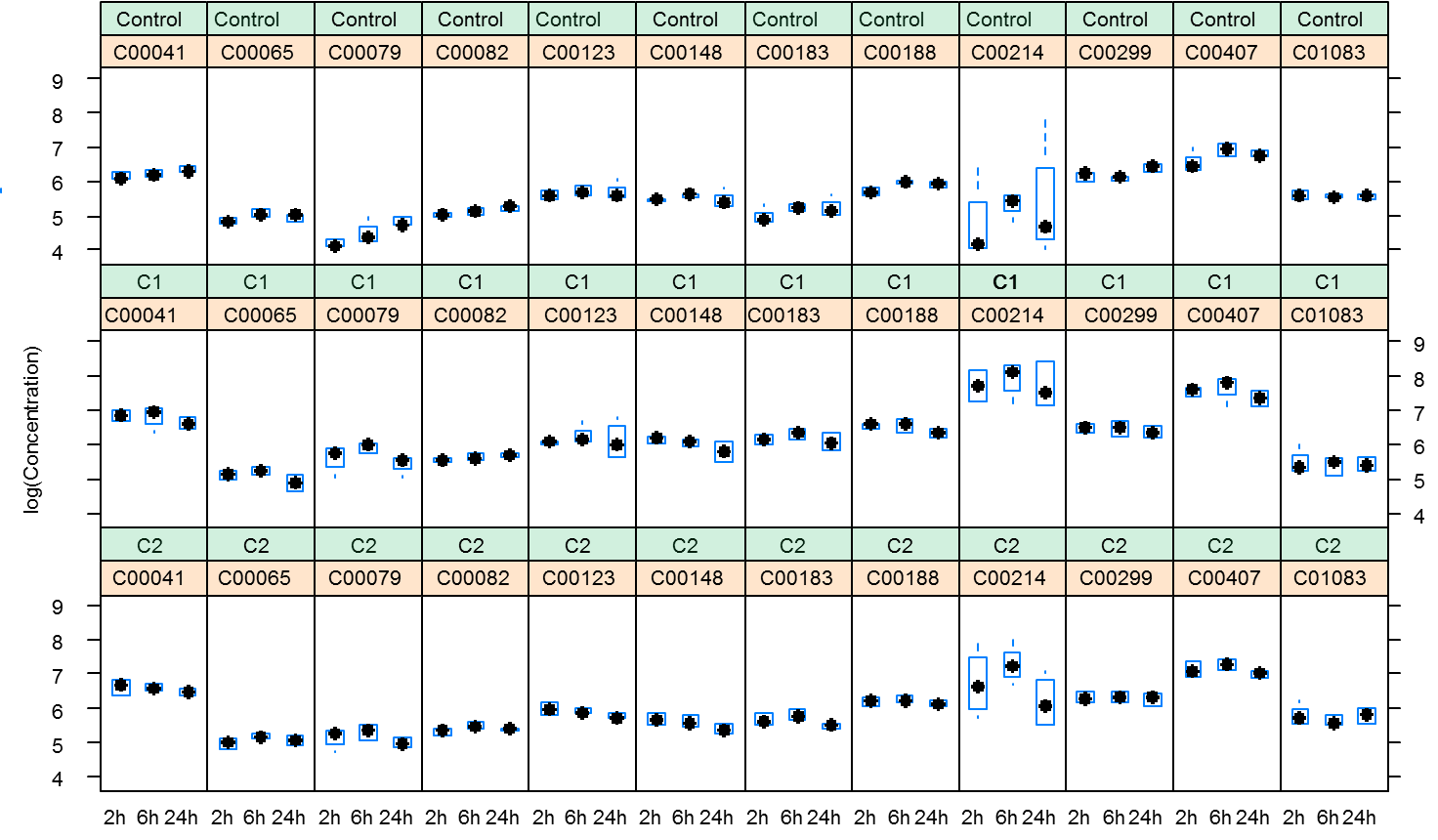


**
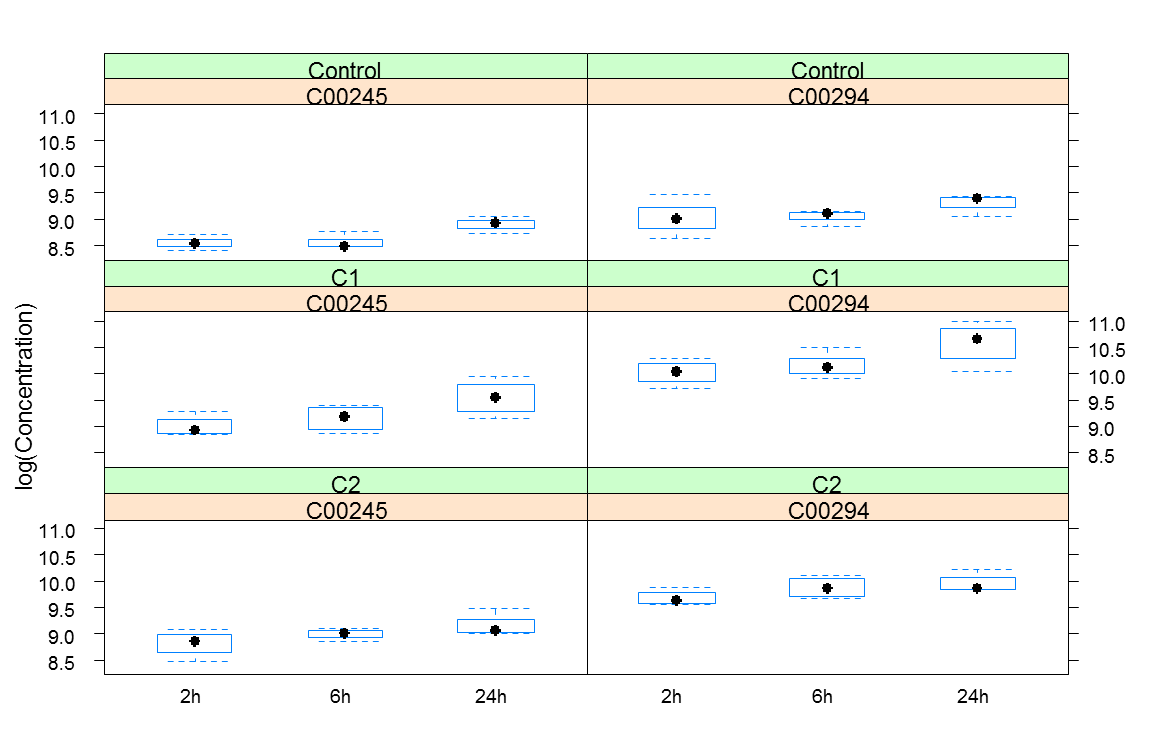
**

**Figure S4.** Lattice graphs representing Triclosan exposures in *G. pulex* at 2, 6 and 24 hours. Representation of controls, low concentration (C_1_) and high concentration (C_2_) in logarithmic scale and using KEGG number notation. Taurine (C00245) and inosine (C00294) are represented in different graphs because of their higher concentrations levels.


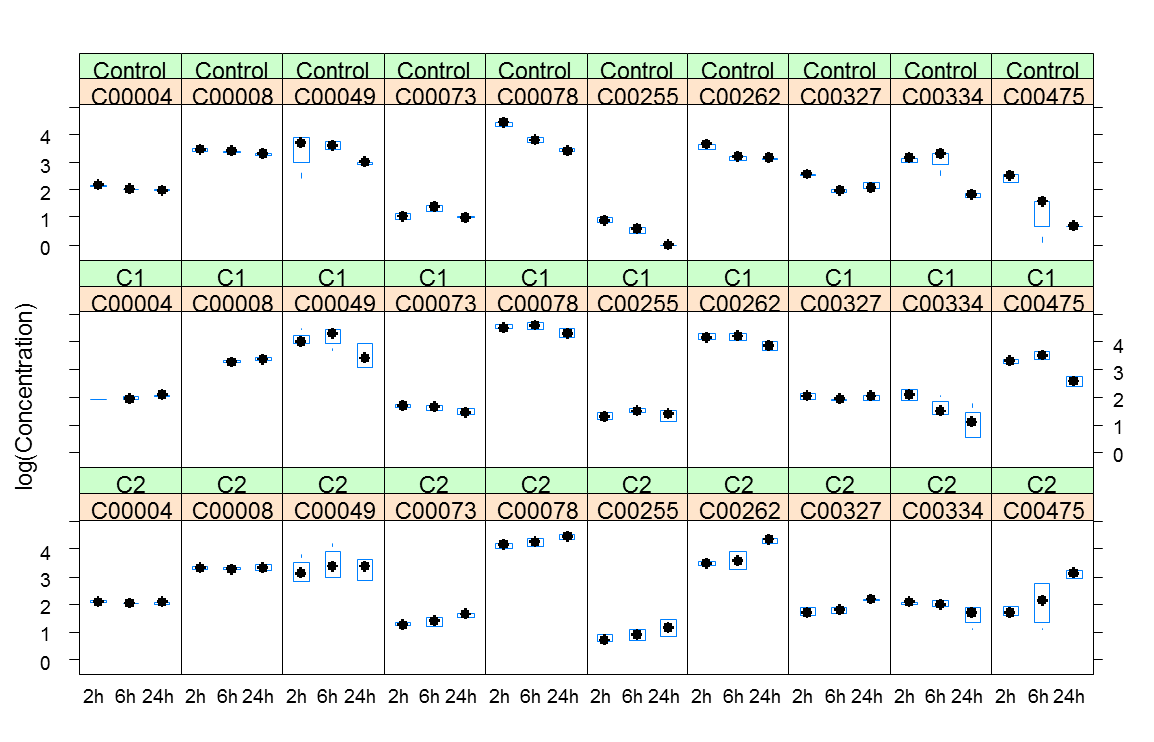


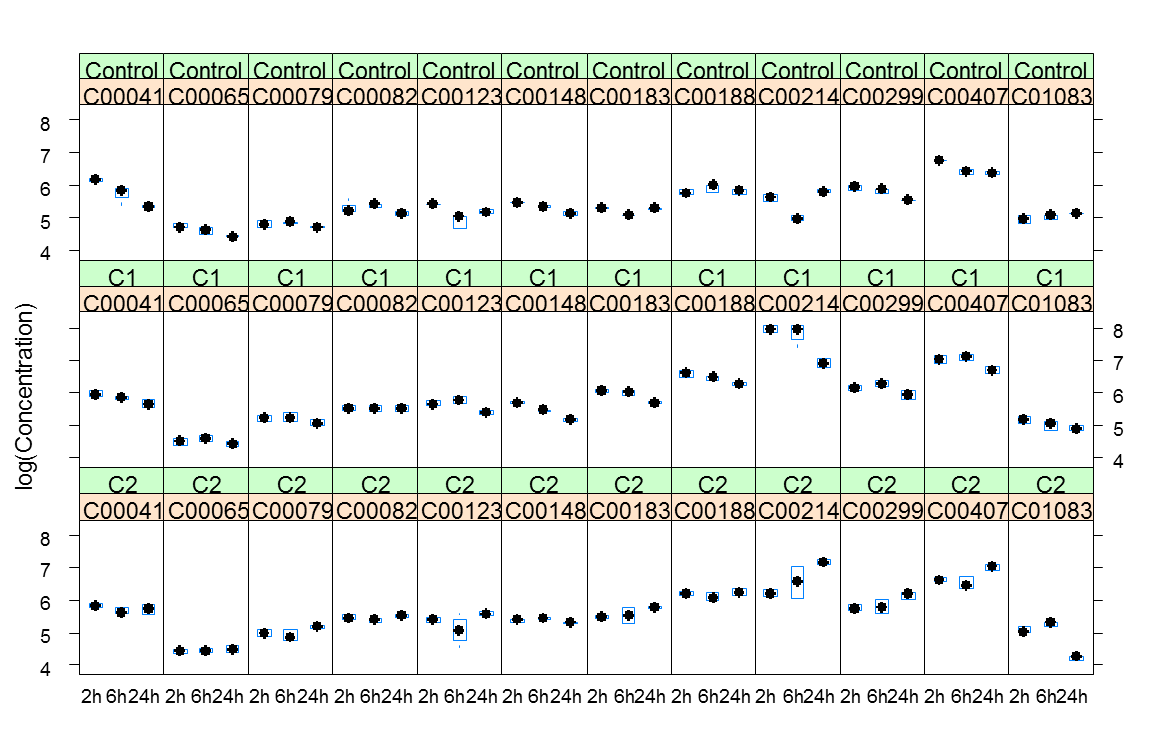


**
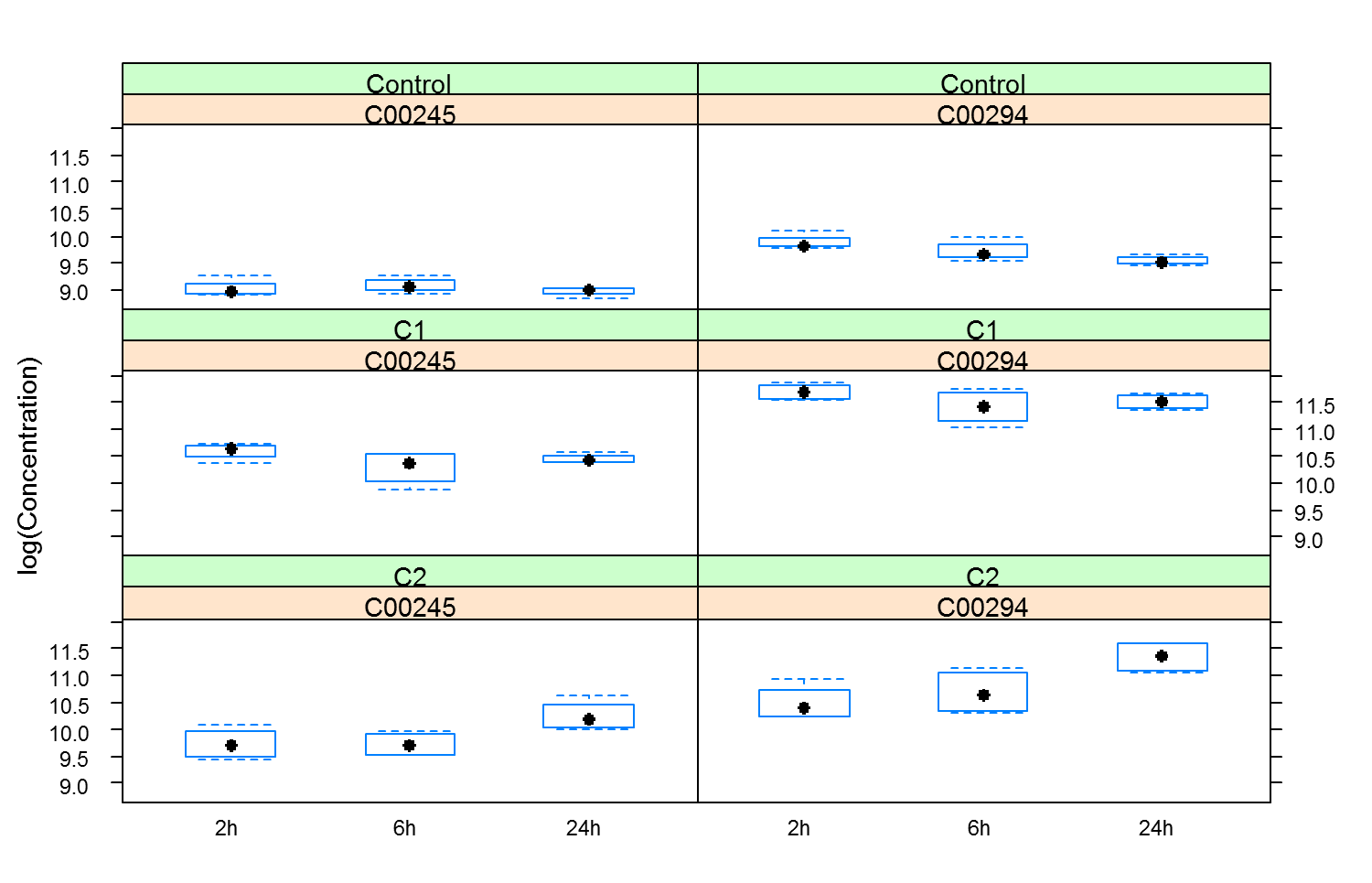
**

**FigureS5.** Lattice graphs representing nimesulide exposures in *G. pulex* at 2, 6 and 24 hours. Representation of controls, low concentration (C_1_) and high concentration (C_2_) in logarithmic scale and using KEGG number notation. Taurine (C00245) and inosine (C00294) are represented in different graphs because of their higher concentrations levels.


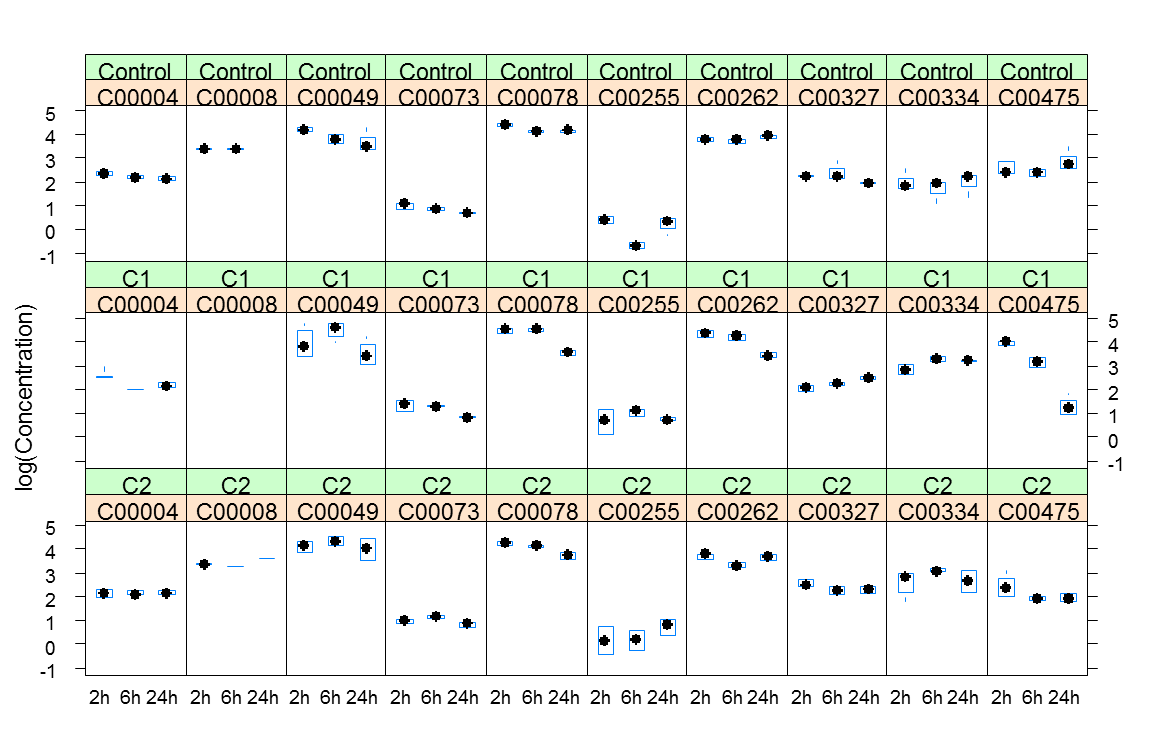


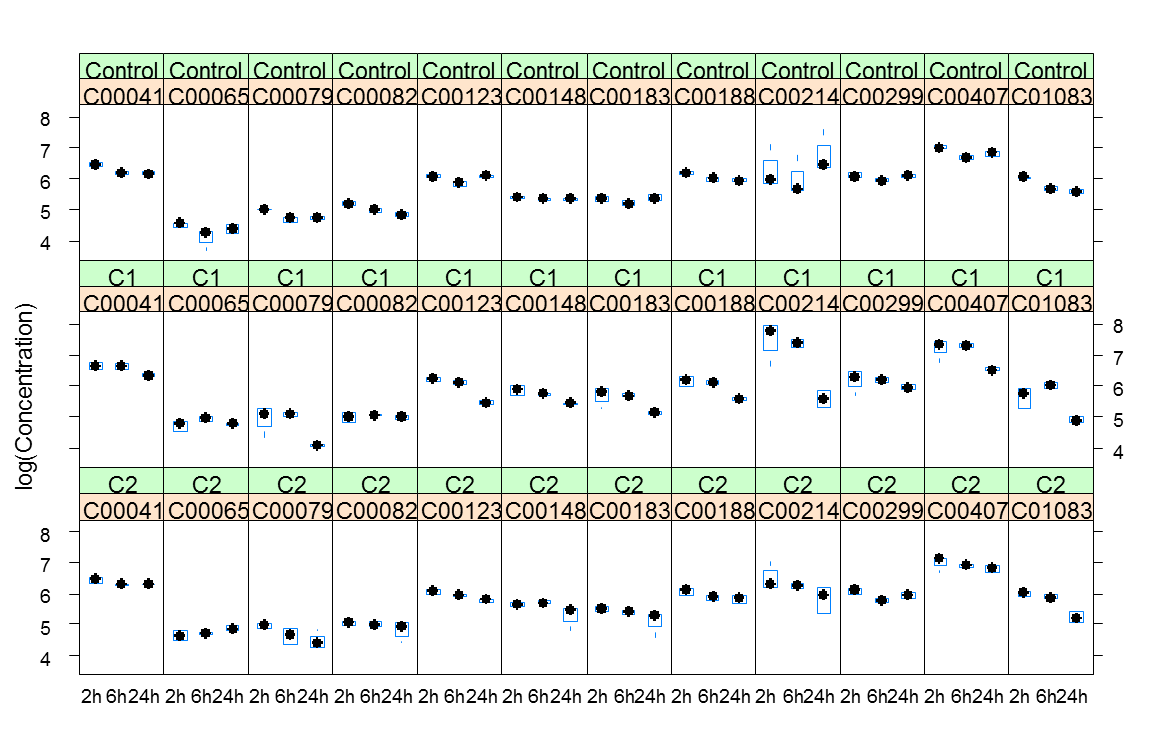


**
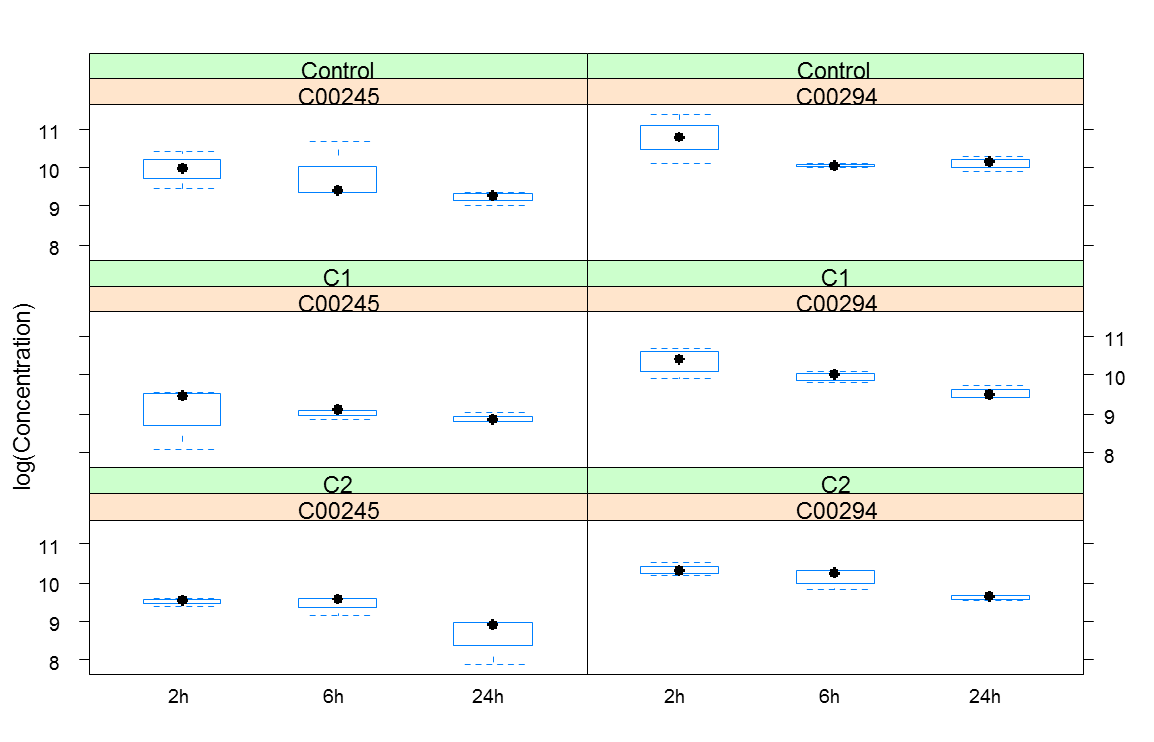
**

**Figure S6.** Lattice graphs representing propanolol exposures in *G. pulex* at 2, 6 and 24 hours. Representation of controls, low concentration (C_1_) and high concentration (C_2_) in logarithmic scale and using KEGG number notation. Taurine (C00245) and inosine (C00294) are represented in different graphs because of their higher concentrations levels.
